# Metabolic reprogramming in the enterocytes of neonatal piglets infected with porcine epidemic diarrhea virus: integrated omics and multi-batch analysis highlight alterations in lipid metabolism and potential therapeutic targets

**DOI:** 10.1128/spectrum.01625-25

**Published:** 2026-04-30

**Authors:** Mengjun Wu, Qian Zhang, Xintao Shi, Peng Li, Zhuan Song, Zhonghua Li, Yanyan Zhang, Lei Wang, Di Zhao, Tao Wu, Dan Yi, Yongqing Hou

**Affiliations:** 1 Hubei Key Laboratory of Animal Nutrition and Feed Science, Wuhan Polytechnic University 74615, Wuhan, China; 2 Engineering Research Center of Feed Protein Resources of Agricultural By-products, Ministry of Education, Wuhan Polytechnic University 74615, Wuhan, China; Barnard College, Columbia University 5798, New York, New York, USA

**Keywords:** porcine epidemic diarrhea virus, piglets, intestinal health, therapeutic targets, lipid metabolism

## Abstract

Porcine epidemic diarrhea (PED) is a severe viral disease caused by the porcine epidemic diarrhea virus (PEDV), leading to huge economic losses in the swine industry. Identifying therapeutic targets has long been a critical challenge in preventing and controlling PED through nutritional interventions. In the present study, 100 seven-day-old crossbred (Duroc × Landrace × Large White) healthy piglets from seven independent trials were selected for the experiment. The transcriptomics, proteomics, and metabolomics analyses were conducted on the small intestine and blood of piglets infected with PEDV, and the combined multiple batches of data were subsequently subjected to integrated analysis. Our findings revealed that PEDV infection significantly affected intestinal cell metabolism, especially lipid metabolism. Among those, sphingolipid and lysophospholipid metabolism could be potential pathways for preventive and therapeutic interventions. Additionally, retinol metabolism, mineral absorption, amino acid metabolism, and pyrimidine metabolism were remarkably altered following PEDV infection. Subsequently, candidate hub genes involved in the core pathways, such as apolipoprotein C3 (APOC3), cytochrome P450 family 3 subfamily A member 22 (CYP3A22), and intestinal alkaline sphingomyelinase (ENPP7), were identified and validated. In conclusion, the present study suggests that PEDV infection leads to the reprogramming of enterocyte lipid metabolism. Furthermore, manipulating lipid metabolism may influence the outcome of viral infection, highlighting potential targets for preventive and therapeutic interventions in managing viral infections.

**IMPORTANCE:**

Porcine epidemic diarrhea (PED) is a severe viral disease caused by the porcine epidemic diarrhea virus (PEDV), leading to huge economic losses in the swine industry. Identifying therapeutic targets has long been a critical challenge in preventing and controlling PED through nutritional interventions. The present study suggests that PEDV infection leads to the reprogramming of enterocyte lipid metabolism. Furthermore, manipulating lipid metabolism may influence the outcome of viral infection. The study also highlights potential targets for preventive and therapeutic interventions in managing viral infections.

## INTRODUCTION

Porcine epidemic diarrhea (PED), caused by the porcine epidemic diarrhea virus (PEDV), is a highly contagious intestinal disease in pigs that leads to vomiting and diarrhea in piglets (1). In recent years, various mutant PEDV strains with higher pathogenicity caused serious PED outbreaks worldwide, posing a significant threat to the global pig industry (2, 3). Previous studies have confirmed that PEDV enters into target cells, specifically the porcine enterocytes, through the fecal-oral and respiratory transmission routes (4). The infection results in dysfunction of intestinal cells and damage to intestinal barriers, as well as induced apoptosis and nutritional malabsorption (5, 6). Currently, the practical strategy for addressing PED primarily relies on palliative interventions due to the lack of effective pharmacological treatments. In addition, the continuous mutation of the PEDV genome and the low efficiency in inducing mucosal immunity pose significant challenges to vaccine-based immunization strategies (7, 8). Therefore, there is an urgent need to identify precise therapeutic targets that can effectively combat PEDV infection in a highly specific manner.

Modern molecular biology technologies, including transcriptomics, proteomics, and metabolomics, are powerful research tools for studying the potential interactions between viruses and their hosts. High-throughput transcriptomic approaches are widely used to study the molecular mechanisms underlying virus-host interactions. RNA-sequencing analysis conducted on pig intestinal cells revealed perturbations in the signal transducer and activator of transcription, cell cycle, apoptosis, and antiviral immune response pathways during PEDV infection (7–10). Proteomic analyses have been widely used to investigate the molecular mechanisms of PEDV infection (11), with most studies conducted using *in vitro* models (12–14). Metabolomics has emerged as a valuable approach for identifying metabolic biomarkers, and when combined with other omics technologies such as proteomics and transcriptomics, it offers an effective framework for understanding biological regulatory networks and identifying potential targets for the prevention and control of infectious diseases (15, 16). During viral infections, viruses can induce protein modifications or metabolic alterations in infected host cells (17). For example, infection with African swine fever virus has been reported to enhance host metabolic pathways, including the tricarboxylic acid cycle and amino acid metabolism, which support viral replication in infected cells (11, 18). Notably, manipulating these changes holds promise for altering the outcomes of viral infections, offering potential targets for prevention and therapeutic interventions (19). However, the metabolic responses to PEDV infection *in vivo*, as well as the key metabolites involved in these processes, are still not well characterized (11). In some *in vitro* models, PEDV-induced metabolic changes have shown that serine, threonine, and methionine metabolism were among the most affected metabolic processes (6). Moreover, limited studies have reported varying metabolomic patterns after PEDV infection, which were mainly associated with mineral absorption (2). Due to unintentional variations in experimental design, platform conditions, reagent lots, operators’ differences, and other non-biological factors, the data obtained from different batches may differ significantly, resulting in misleading outcomes (20). There is an urgent need for large-scale omics data sets with multiple batches to provide robust, stable, and reliable insights into PEDV infection in piglets when analyzed through a systems biology framework. To the best of our knowledge, no public studies have reported the results of integrated omics and multi-batch analyses regarding PEDV infection in neonatal piglets.

Therefore, in the present study, we provide a systematic view of the transcriptome, proteome, and metabolic profiles in infected piglets by integrating multi-analyses and multi-omics approaches, which uncover the complex host response to PEDV. In addition, we identified and validated prospective targets for both diagnostic and therapeutic purposes in the context of PEDV. Importantly, this study offers a novel perspective for understanding the pathogenesis of PEDV and provides new insights into its prevention and control.

## MATERIALS AND METHODS

### Animal experiment

**Fig 1:**
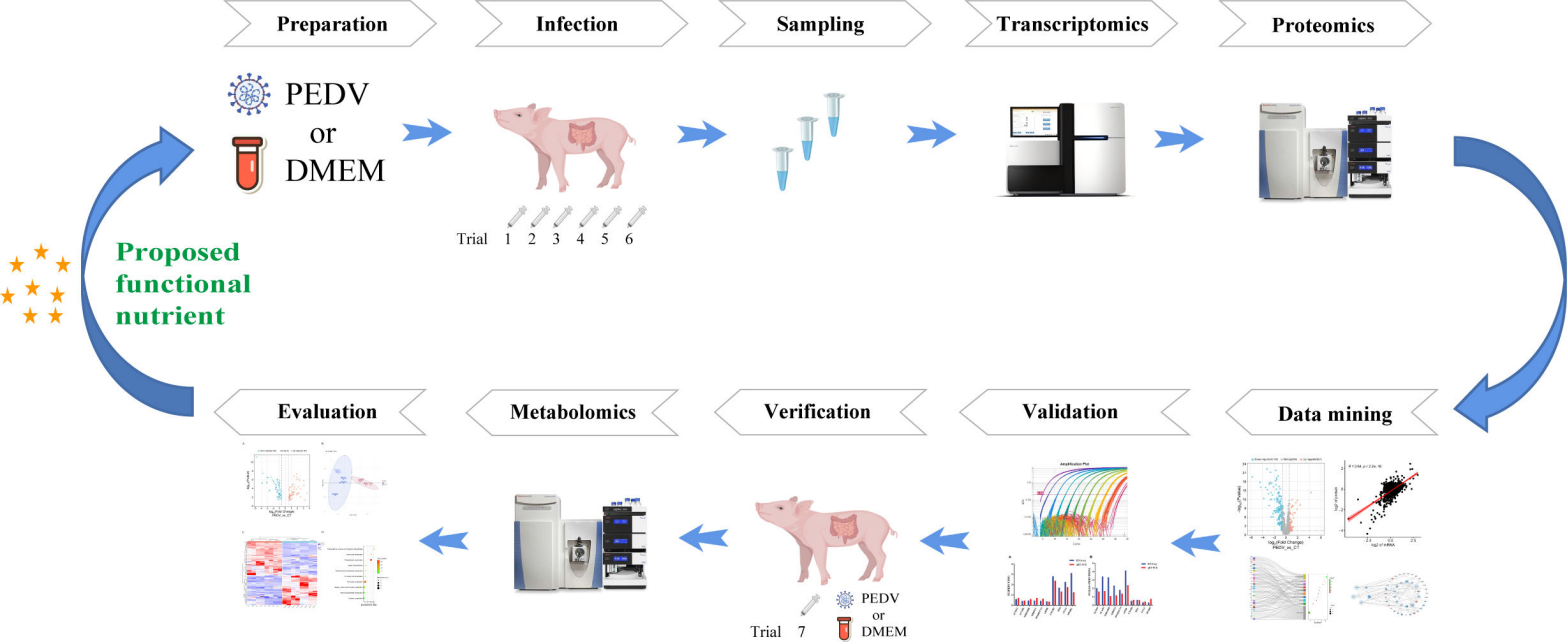
Graphical workflow illustrating the methodology employed in the present study.

In total, 100 seven-day-old crossbred (Duroc × Landrace × Large White) healthy piglets from seven independent trials were selected and randomly allocated to either the control group or the PEDV group. The PEDV YN13 strain, a member of GIIb strains, was isolated and purified from the jejunum of a diarrheal pig in 2015 and stored in our laboratory. The viral inoculum was prepared from cell culture and quantified by TCID_50_. The quantification methods inherently involve experimental variation. Therefore, reporting the dose as a range reflects the real biological and technical uncertainty in viral titration. Secondly, even within the same viral stock, small fluctuations occur between batches or aliquots in infectious particle concentration or viability after thawing. To maintain transparency and reproducibility, the dose is reported as a range that encompasses the measured titers of the inoculum used for infection. In addition, the goal of the infection model is to achieve uniform clinical disease without excessive mortality. The range allows us to adjust for slight differences in virus stock potency while maintaining comparable pathogenic effects among experiments or replicates. Before each formal trial, we conduct a preliminary experiment to determine the TCID_50_. In summary, the infection dose was expressed as a range rather than a fixed value to account for the natural variability in viral titration and inoculum preparation, to ensure reproducibility of infection severity across piglets, and to accurately reflect the precision limits of TCID_50_ quantification methods. After a 5-day adaptation period, piglets in the PEDV group received oral administration of PEDV (GenBank accession No. KT021228) at a dose from 10^4.5^ TCID_50_ to 10^6.0^ TCID_50_, while those in the control group received an equivalent volume of DMEM as the mock control. Our research group has previously established a validated PEDV-induced intestinal injury model in piglets, in which clinical symptoms appear at 24 h post-infection and peak at approximately 48 h, accompanied by severe intestinal damage and active viral replication (5, 19). Accordingly, 2 days post-infection, blood was collected, and piglets were anesthetized with a pentobarbital sodium injection (50 mg/kg BW). Subsequently, intestinal samples were separated at 1/2 of the jejunum and ileum. The samples were collected after being cleaned with ice-cold saline, then rapidly frozen in liquid nitrogen and stored at −80°C for further analysis. A graphical workflow illustrating the methodology employed in the present study is shown in Fig. 1.

### Feeding management

Pig pens, walls, and floors in the animal house were thoroughly cleaned, disinfected, and fumigated before the animal experiment. The environmental conditions were controlled at 26–28°C, humidity 50–60%, with appropriate ventilation during the animal experiment. Piglets from different groups were housed in separate rooms to prevent cross-infection. A basal diet (liquid milk substitute, dry matter content 20%) was formulated to meet the nutritional needs of suckling piglets, and the liquid diet (45°C–55°C) was supplied every 3 h from 8 a.m. to 8 p.m. The house was cleaned twice daily using alternative disinfectants (povidone-iodine, glutaraldehyde, and benzalkonium bromide). Piglets were observed and recorded daily for behavior, general health, diarrhea, and clinical symptoms.

### Intestinal morphology

Intestinal histomorphology was assessed following the methodology described previously (21). Small intestine samples, each 1 cm long, were fixed in 4% paraformaldehyde. The fixed samples were then dehydrated, embedded in paraffin, sectioned at a thickness of 4 μm, and stained with hematoxylin and eosin. Morphological examination was conducted using a light microscope (Leica Microsystems, Wetzlar, Germany) in conjunction with the Leica Application Suite image analysis software (Leica Microsystems, Wetzlar, Germany).

### RNA isolation and quantitative real-time PCR

Jejunal and ileal samples (100 mg) were placed in 2 mL centrifuge tubes, and RNA was extracted using RNAiso Plus reagent (Takara, Dalian, China) according to the manufacturer’s instructions (5). Total RNA integrity was assessed by electrophoresis on a 1% agarose gel. Then, RNA purity was evaluated by measuring the absorbance ratios at 260/280 nm and 260/230 nm using a NanoDrop spectrophotometer (Thermo Fisher Scientific, USA). Samples with A260/280 ratios between 1.8 and 2.0 and A260/230 ratios between 1.8 and 2.2 were considered to be of high purity and suitable for downstream analyses. Then, cDNA was synthesized using the PrimeScript RT reagent kit with gDNA Eraser (Takara, Dalian, China). Quantitative real-time PCR (qRT-PCR) was performed using the Taq Pro Universal SYBR qPCR Master Mix (Vazyme, Nanjing, China) on an Applied Biosystems 7500 Fast qRT-PCR System (Foster City, CA, USA) under the following conditions: 95°C, 30 s; 95°C, 5 s; 60°C, 34 s (40 cycles); 95°C, 15 s; 60°C, 1 min; 95°C, 15 s. The primers of the PEDV-M and PEDV-N genes were reported in previous publications (22, 23). Results were expressed as CT values representing the expression of the viral load in the samples (24). Additionally, the selected hub genes were validated using RT-qPCR. The primer sequences used in the present study are listed in Table 1.

**TABLE 1:** Sequence of the oligonucleotide primers used in present study^*a*^

Gene	Primer sequences (5′→ 3′)	NCBI accession number
PEDV-M	F:TCCCGTTGATGAGGTGAT	NC_003436.1
	R:AGGATGCTGAAAGCGAAAA	
PEDV-N	F:CGCAAAGACTGAACCCACTAACTT	NC_003436.1
	R:TTGCCTCTGTTGTTACTCGGGGAT	
APOA1	F: CCTTGGCTGTGCTCTTCCTC	NM_214398.1
	R: ACGGTGGCAAAATCCTTCAC	
APOB	F: GGGATGATGGCACAGGTTACA	NM_001375388.1
	R: TGACGTGGACTTGGTGCTTT	
APOC3	F: CTAACCAGCGTGAAGGAGTC	NM_001002801.1
	R: CAGAAGTCGGTGAACTTGCC	
ARG1	F: GGCTGGTCTGCTTGAGAAAC	NM_214048.2
	R: ATCGCCATACTGTGGTCTCC	
ASAH2	F: ATAGAGCACCTACAGGCAAAC	XM_021072693.1
	R: TCGGGTTAGCACCTACAAATAC	
CYP3A22	F: AAACGGACGCCCATAAAG	NM_001195509.1
	R: GGGAAGGTCGCATCAATCTC	
ENPP7	F: CTGCCTTATCACACCACACT	XM_013980746.2
	R: CGCCTTGGTAGGTGACATT	
FABP2	F: GAAACTTGCAGCTCATGACAAT	NM_001031780.1
	R: GTCTGCGAGGCTGTAGTTAAA	
HSD17B6	F: AAGACTGAGACCTGGATACATACC	XM_003355459.4
	R: GACAATTCTCCCCTGTGCTT	
KCNJ13	F: ATGGATGTGTCGCTGGTCTTT	XM_001926506.6
	R: CACAACTGCTTGCCTTTACGAG	
MEP1A	F: AAGCTGGTCAAGATGAAGACCT	XM_021098804.1
	R: TTTGAGTTCTGGGGATCACCTT	
MME	F: CACAACATCAGAAACAGCGACA	XM_021069692.1
	R: GGCAATCAAATCCTCAACCAC	
PCK1	F: CGGGATTTCGTGGAGA	NM_001123158.1
	R: CCTCTTGATGACACCCTCT	
PLA2G3	F: ACTCTGCTGGGAACTCATCT	XM_003132975.4
	R: GGTAGTTTCGGATGCCATAGTT	
SCARB1	F: TCCCTGTCCCTTTCTACCTC	NM_213967.1
	R: CTCTTGTGCCTGAACTCCCT	
SLC27A2	F: TTTTCAGCCAGCCACTTTTG	NM_001278777.1
	R: CATTTGGTTTCTGGGGAGAGTT	
TRPM6	F: TACGGGAAGAGATGTGGTGT	XM_021064975.1
	R: CGCCTGAGCTTCATCTCATT	
TRPV6	F: AGGAGCTGGTGAGCCTCAAGT	XM_021078898.1
	R: GGGGTCAGTTTGGTTGTTGG	
ENPP3	F: CTATCCAACCAAAACCTTCCC	XM_021087960.1
	R: GCTGGATTTTCTTTCTCCTTCG	
CYP2J34	F: TGAGGCTGTTGGATGAAGTC	NM_001244633.1
	R: TGAAGAGGGTTTGGTGGG	
ENTPD8	F: GCTCATCCTGATCCTCGTG	XM_021081094.1
	R: GTGTCGTTCTCCTTGTTTGC	
TK1	F: GATTGCCCAGTACAAGTGCC	XM_021066553.1
	R: TCCCGAAAGCCTTCCTCT	

^*a*^ PEDV-M, membrane protein (porcine epidemic diarrhea virus); PEDV-N, nucleocapsid protein (porcine epidemic diarrhea virus); APOA1, apolipoprotein A1; APOB, apolipoprotein B; APOC3, apolipoprotein C3; ARG1, arginase 1; ASAH2, N-acylsphingosine amidohydrolase 2; CYP3A22, cytochrome P450 family 3 subfamily A member 22; ENPP7, ectonucleotide pyrophosphatase/phosphodiesterase 7; FABP2, fatty acid binding protein 2; HSD17B6, hydroxysteroid 17-beta dehydrogenase 6; KCNJ13, potassium inwardly rectifying channel subfamily J member 13; MEP1A, meprin A subunit alpha; MME, membrane metalloendopeptidase; PCK1, phosphoenolpyruvate carboxykinase 1; PLA2G3, phospholipase A2 group III; SCARB1, scavenger receptor class B member 1; SLC27A2, solute carrier family 27 member 2; TRPM6, transient receptor potential cation channel subfamily M member 6; TRPV6, transient receptor potential cation channel subfamily V member 6; ENPP3, ectonucleotide pyrophosphatase/phosphodiesterase 3; CYP2J34, cytochrome P450 family 2 subfamily J member 34; ENTPD8, ectonucleoside triphosphate diphosphohydrolase 8; TK1, thymidine kinase 1.

### Transcriptome sequencing and analysis

Briefly, RNA was extracted using TRIzol reagent (Invitrogen, Carlsbad, CA, USA), following the manufacturer’s instructions. The integrity and purity of the total RNA were checked before the construction of the sequencing library. The NEBNext Ultra RNA Library Prep Kit for Illumina (New England Biolabs, Ipswich, MA, USA) was used to generate the sequencing libraries. After purification, they were subjected to deep sequencing on an Illumina Hiseq platform (Illumina, San Diego, CA, USA) (25, 26). Gene expression levels were normalized, and differentially expressed genes (DEGs) were identified with a fold change ≥3 or ≤0.33 and an adjusted *P*-value < 0.05. DEGs were subjected to Gene Ontology (GO), Kyoto Encyclopedia of Genes and Genomes (KEGG), and Reactome pathway enrichment analyses using the Database for Annotation, Visualization and Integrated Discovery (DAVID) website (https://david.ncifcrf.gov/) (27). Furthermore, protein-protein interaction networks were constructed using the STRING online database (https://string-db.org/) in combination with Cytoscape software (v.3.7.1, https://cytoscape.org/), and hub genes were identified using Cytoscape software with the CytoHubba plugin (28).

### Label-free proteomic analysis using data-dependent acquisition mass spectrometry (DDA-MS)

The label-free quantitative proteomic analysis was conducted in these studies. In short, the intestinal tissue was homogenized with Tissue Protein Extraction Reagent (Thermo Scientific, USA) containing protease inhibitors (Roche, USA) and then vortexed for 10 min at 4°C. The soluble protein was collected by centrifugation (12,000 × *g*, 20 min, 4°C) and quantified using the bicinchoninic acid (BCA) assay. Peptides were generated using the filter-aided sample preparation (FASP) trypsin digestion method, as previously reported (24). The quantified peptides were further analyzed on a Q Exactive mass spectrometer (Thermo Fisher Scientific, USA) coupled to an Easy-Nano Ultimate 3000 UPLC system (Dionex, Thermo Fisher Scientific, USA). The peptides were loaded into a trap column (100 μm × 20 mm; Acclaim PepMap 100; Thermo Fisher Scientific, USA) and then separated on a C18 capillary column (75 μm × 150 mm; Acclaim PepMap RSLC; Thermo Fisher Scientific, USA) within 125 min. The peak lists extracted from raw mass spectrometry (MS) data were further processed using MaxQuant software (version 1.6.1.0, Max Planck Institute of Biochemistry, Martinsried, Germany) with the extracted ion chromatogram (XIC)-based label-free quantitation algorithm. Protein abundance was normalized, and differentially expressed proteins (DEPs) were identified with a fold change ≥2 or ≤0.5 and an adjusted *P*-value < 0.05. The procedure for bioinformatics analysis followed the same approach as described in the transcriptomic analysis.

### Integrated analyses of transcriptomic and proteomic data

First, a Venn diagram was generated to illustrate the relationship between transcriptomic and proteomic results. In order to examine the congruence between transcriptomics and proteomics, a correlation analysis was performed between DEGs and DEPs data, as indicated by a scatter plot representing the correlation between proteins and transcripts. Overlapping genes/proteins with the same expression trend were selected for further GO, KEGG, and Reactome pathway analyses. In addition, protein-protein interaction networks were constructed using the STRING online database (https://string-db.org/) in combination with Cytoscape software (v.3.7.1, https://cytoscape.org/).

### Untargeted HPLC/MS-based metabolomics

Metabolites were extracted from blood samples using a methanol:water (4:1, vol/vol) solution. An HSS T3 column (100 mm × 2.1 mm i.d., 1.8 μm) was employed to separate samples with mobile phases consisting of 0.1% formic acid in water:acetonitrile (95:5, vol/vol) and 0.1% formic acid in acetonitrile:isopropanol:water (47.5:47.5:5, vol/vol). The conditions for the Thermo UHPLC-Q Exactive HF-X Mass Spectrometer were as follows: heater temperature, 425℃; capillary temperature, 325°C; sheath gas flow rate, 50 arb; aux gas flow rate, 13 arb; ion-spray voltage floating (ISVF), −3,500 V in negative mode and 3,500 V in positive mode; and normalized collision energy, with a rolling setup of 20–40–60 V for MS/MS. Full MS resolution was set at 60,000, and MS/MS resolution was set at 7,500. Data acquisition was conducted in Data Dependent Acquisition (DDA) mode over a mass range of 70–1,050 m/z. The raw LC/MS data were preprocessed with Progenesis QI (Waters Corporation, Milford, USA), and metabolites were identified using the HMDB, METLIN, and Majorbio databases.

The raw data were then analyzed using the Majorbio Cloud platform (cloud.majorbio.com) (29). The stability of the model was evaluated using the R package “ropls” (Version 1.6.2) to perform principal component analysis (PCA) and orthogonal least partial squares discriminant analysis (OPLS-DA) (30). Moreover, Student’s *t*-test and fold change analysis were executed, combined with variable importance in the projection (VIP) obtained from the OPLS-DA model, to identify the significantly different metabolites (VIP > 1, *P* < 0.05, fold change ≥1.5 or ≤0.67) (31). Differential metabolites between groups were mapped to their biochemical pathways using KEGG database searches, and statistically significant pathways were identified using Fisher’s exact test.

### Blood biochemical measurements

The collected blood was centrifuged at 3,000 × *g* at 4°C for 10 min to prepare plasma and stored at −80°C for the assay of biochemical parameters. In short, the concentrations of blood lipid-related indexes, including total cholesterol (TC), triglyceride (TG), high-density lipoprotein (HDL), and low-density lipoprotein (LDL) in blood, were quantified with a Hitachi 7060 Automatic Biochemical Analyzer (Hitachi, Japan).

### Statistical analysis

Data from RT-qPCR for selected hub genes and blood biochemical measurements were analyzed using a *t*-test with SPSS 26.0 (SPSS, Inc., Chicago, IL, USA) and plotted using PRISM (GraphPad Software Inc., San Diego, CA, USA). The results are presented as the mean ± standard deviation (SD). *P* < 0.05 was considered statistically significant.

## RESULTS

### Establishment of intestinal injury model in piglets with PEDV infection

**Fig 2:**
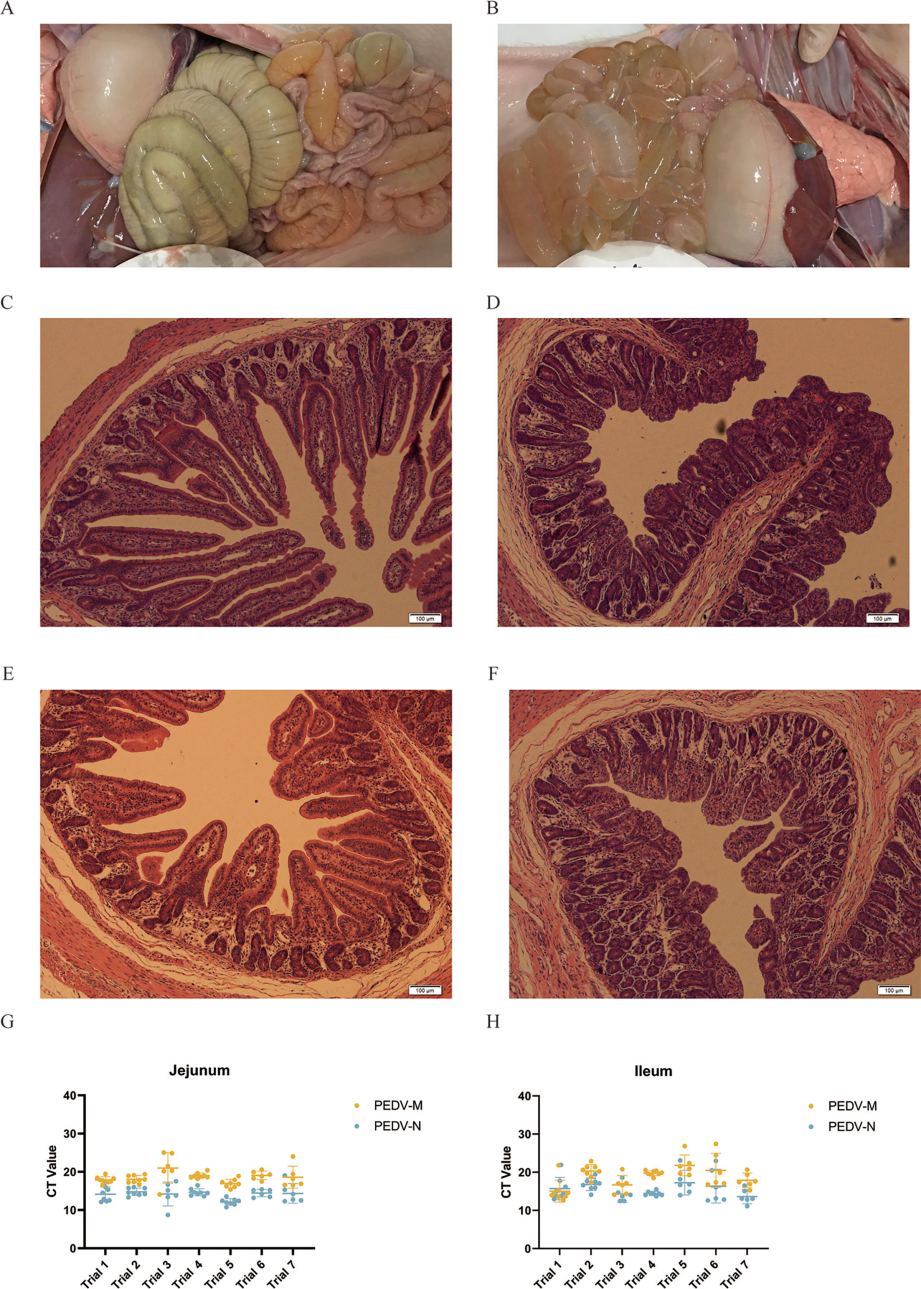
Effects of PEDV infection on clinical symptoms and intestinal histopathology in piglets. (**A**) On-site anatomy of piglets in the control (CT) group. (**B**) On-site anatomy of piglets in the PEDV group. (**C**) Histopathological structures of piglet jejunum in the CT group. (**D**) Histopathological structures of piglet jejunum in the PEDV group. (**E**) Histopathological structures of piglet ileum in the CT group. (**F**) Histopathological structures of ileum in the PEDV group. (**G**) Original CT values of PEDV-M and PEDV-N genes in the jejunum of piglets in the PEDV group. (**H**) Original CT values of PEDV-M and PEDV-N genes in the ileum of piglets in the PEDV group.

After infection, the piglets in the PEDV group exhibited severe diarrhea, and clinical necropsies revealed that the small intestinal walls of these piglets were thin, transparent, and filled with watery liquid (Fig. 2B). Tissue sections stained with H&E showed intestinal histopathological changes in the infected group, with damage to the small intestinal villi, lysis, and necrosis of the intestinal epithelial cells, which were detached from the underlying tissue (Fig. 2D through F). RT-qPCR analysis of intestinal segments indicated that PEDV effectively replicated in the small intestine of piglets (Fig. 2G and H).

### Transcriptomic analysis of the small intestine in PEDV-infected piglets

**Fig 3:**
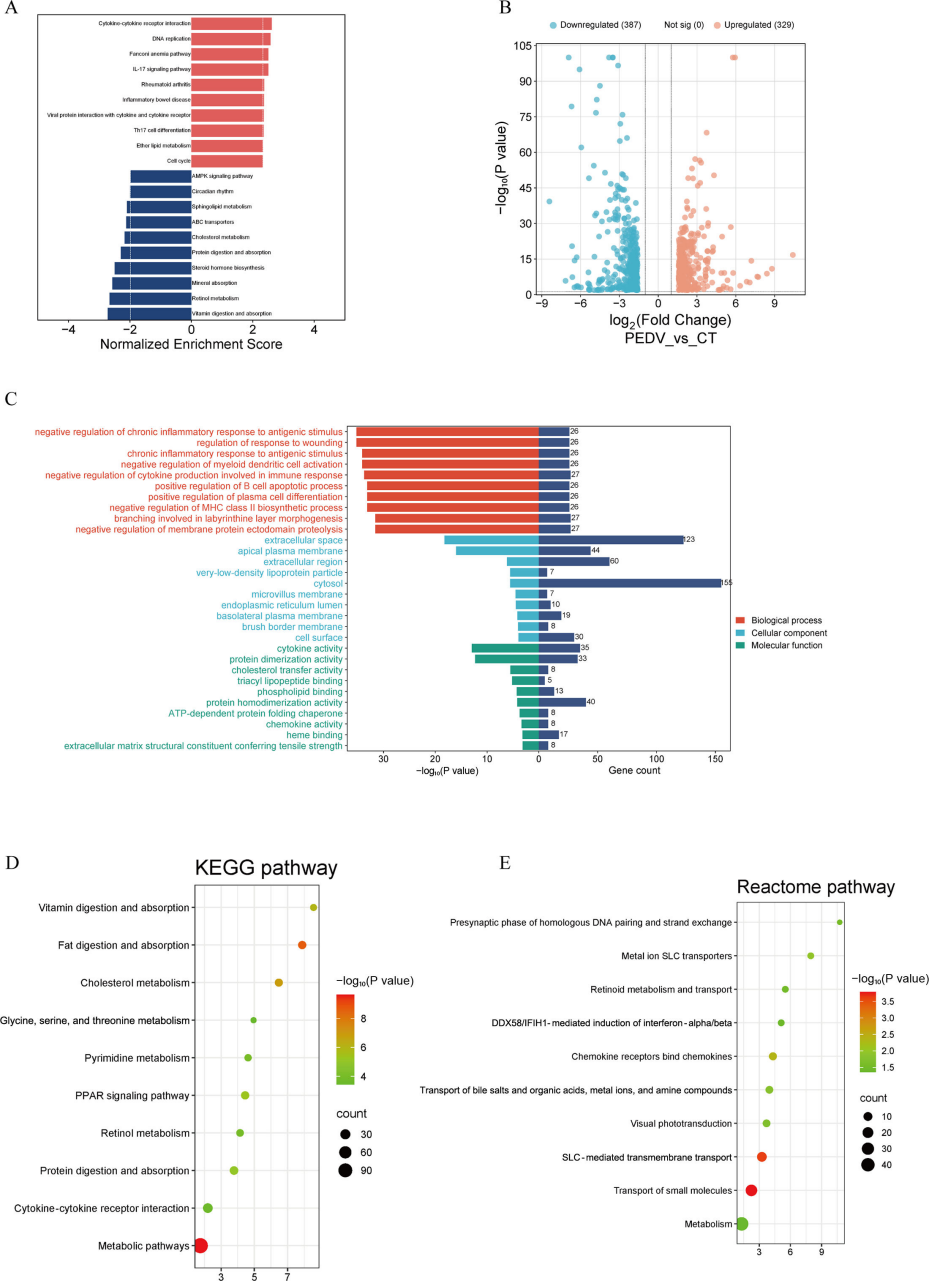
Transcriptomic analysis of the small intestine in PEDV-infected piglets from trial 1. (**A**) Gene set enrichment analysis (GSEA). (**B**) Volcano plot depicting differential expression. (**C**) Gene Ontology (GO) analysis. (**D**) KEGG pathway analysis. (**E**) Reactome pathway analysis.

**Fig 4:**
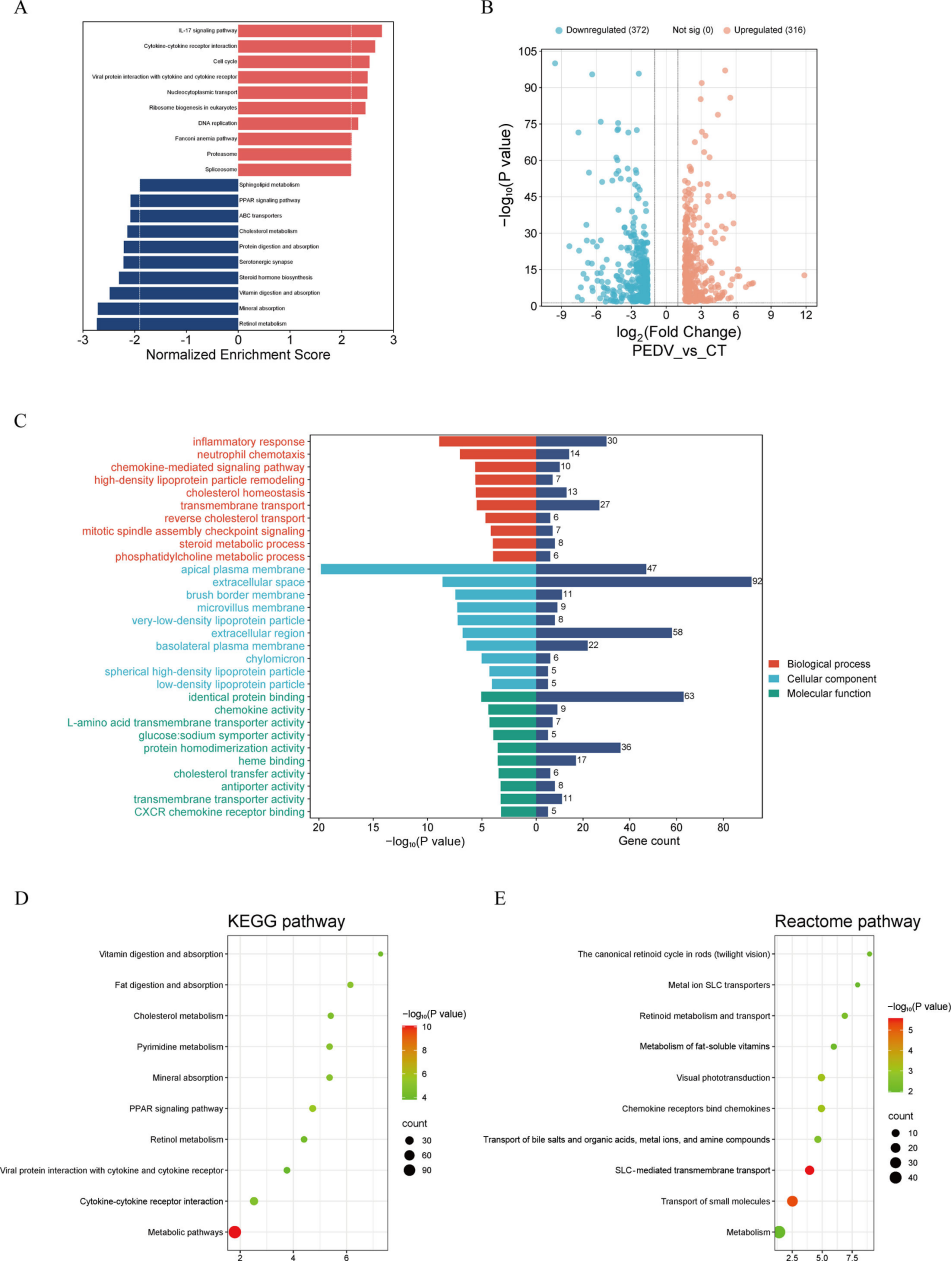
Transcriptomic analysis of the small intestine in PEDV-infected piglets from trial 2. (**A**) GSEA. (**B**) Volcano plot. (**C**) GO analysis. (**D**) KEGG pathway analysis. (**E**) Reactome pathway analysis.

**Fig 5:**
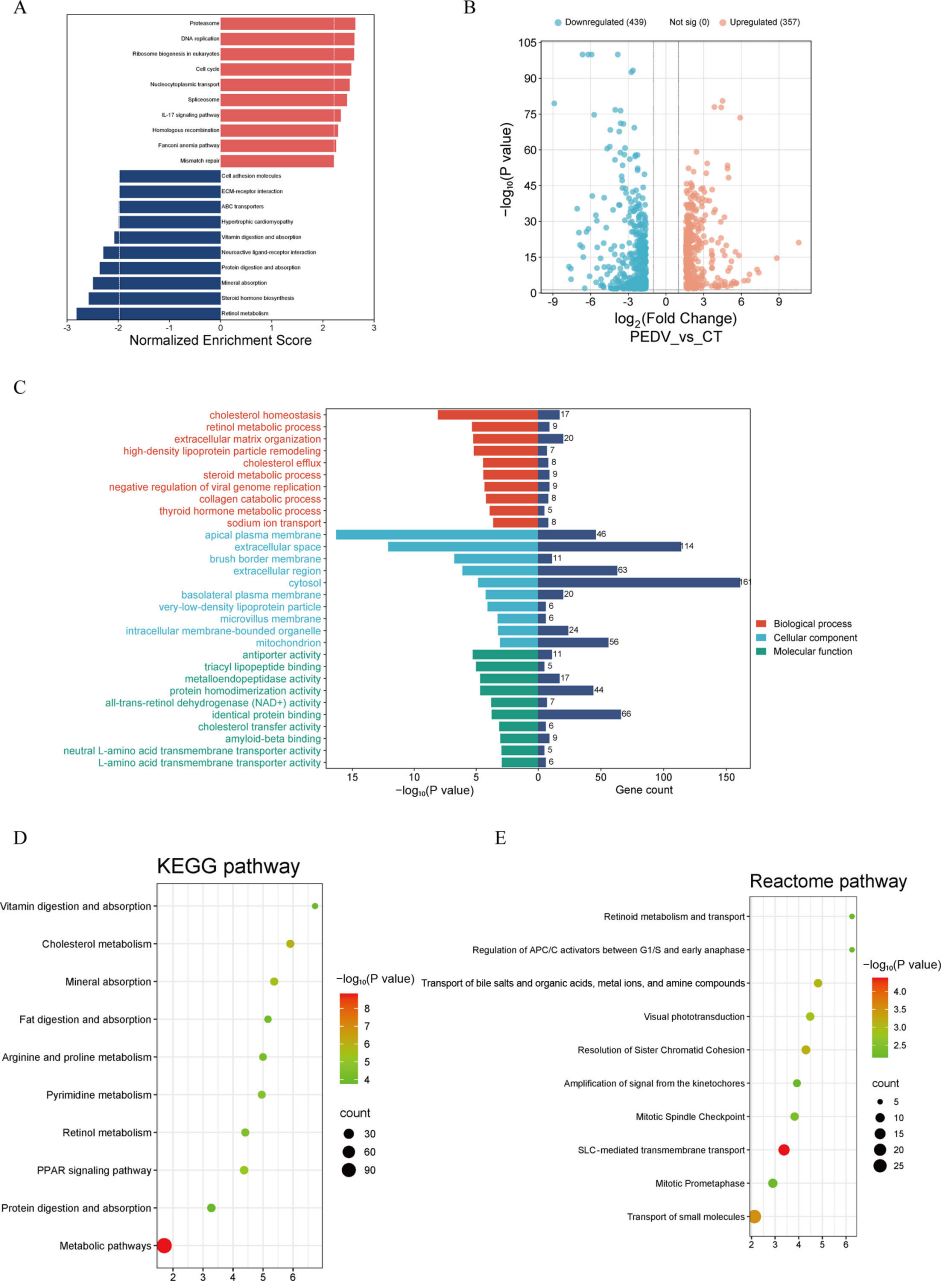
Transcriptomic analysis of the small intestine in PEDV-infected piglets from trial 3. (**A**) GSEA. (**B**) Volcano plot. (**C**) GO analysis. (**D**) KEGG pathway analysis. (**E**) Reactome pathway analysis.

To obtain a comprehensive understanding of the host transcriptional response to PEDV infection, we performed an integrated analysis of all three transcriptomic trials using both GSEA and DEG-based functional enrichment. Across the three independent experiments, GSEA consistently revealed that pro-inflammatory and immune-related pathways—particularly cytokine–cytokine receptor interaction and the IL-17 signaling pathway—were positively enriched in PEDV-challenged piglets. In contrast, pathways associated with nutrient metabolism and intestinal absorptive function, such as retinol metabolism, vitamin, and fat digestion and absorption, and mineral absorption, were repeatedly and robustly negatively enriched (Fig. 3A; Fig. 4A; Fig. 5A). Differential expression analysis further supported these findings, with 688–796 DEGs identified per trial and a highly comparable number of up- and downregulated genes across experiments (Fig. 3B; Fig. 4B; Fig. 5B). Functional annotation of these DEGs revealed consistent enrichment of biological processes related to immune activation, lipid and retinoid metabolic processes, and transmembrane transport (Fig. 3C; Fig. 4C; Fig. 5C). KEGG pathway enrichment further highlighted alterations in cholesterol, retinol, and pyrimidine metabolism in all three trials, indicating a shared metabolic reprogramming pattern induced by PEDV infection (Fig. 3D; Fig. 4D; Fig. 5D). Reactome analysis also recurrently identified retinoid metabolism and transport of small molecules as significantly affected pathways across data sets (Fig. 3E; Fig. 4E; Fig. 5E). Protein-protein interaction network analysis identified 20 hub genes in each trial (Fig. S1, S2, and S3), collectively suggesting that PEDV infection induces a host response characterized by heightened inflammation, disrupted epithelial nutrient absorption, and extensive metabolic dysregulation—particularly involving lipid and retinoid pathways.

### Proteomic analysis of small intestine in PEDV-infected piglets

**Fig 6:**
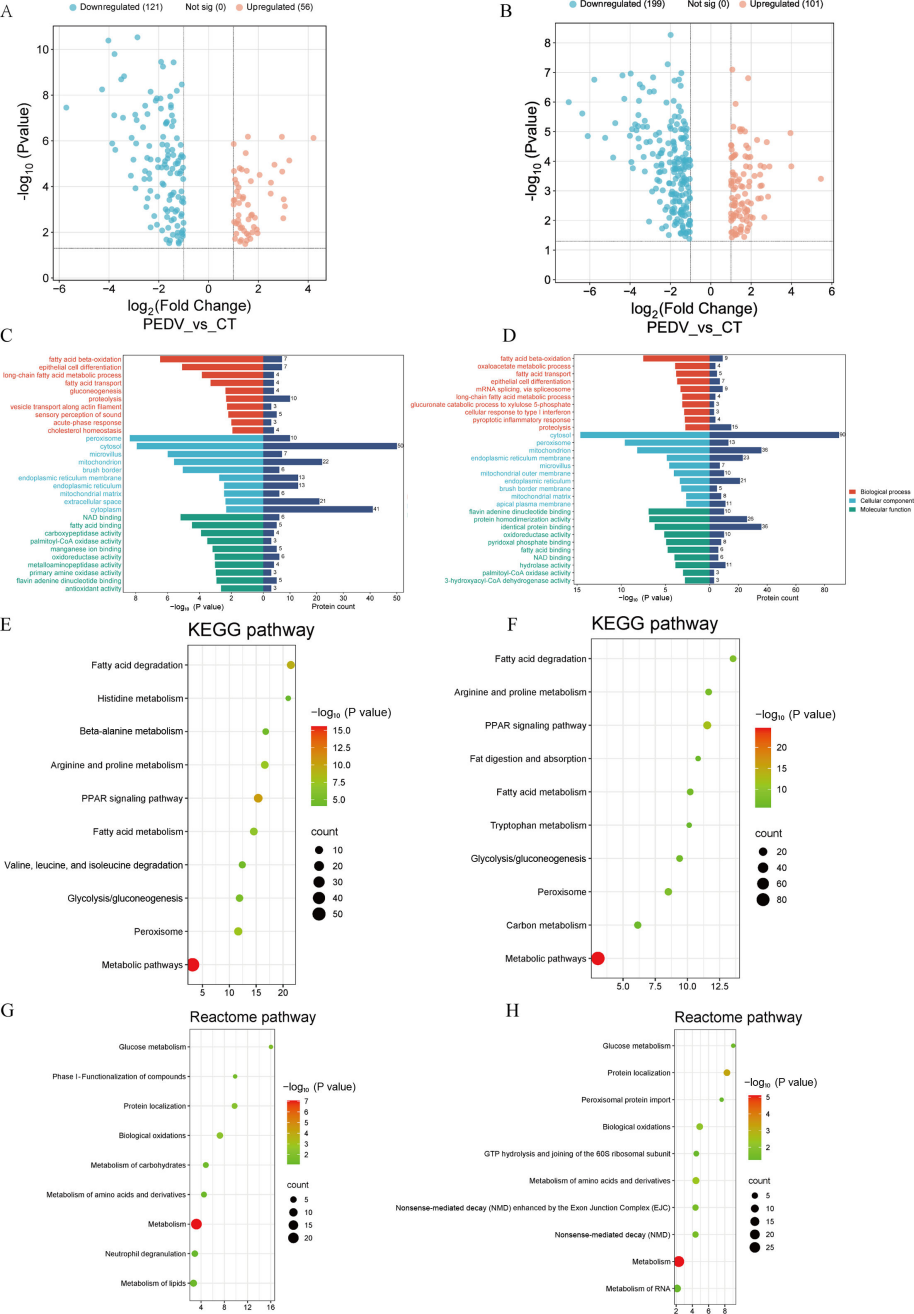
Proteomic analysis of the small intestine in PEDV-infected piglets from trials 4 (**A, C, E, G**) and 5 (**B, D, F, H**). (**A and B**) Volcano plot. (**C and D**) GO analysis. (**E and F**) KEGG pathway analysis. (**G and H**) Reactome pathway analysis.

To further elucidate the host metabolic alterations induced by PEDV infection, we integrated the proteomic results from trials 4 and 5. Across both experiments, PEDV challenge led to substantial changes in protein expression, with 177-300 DEPs identified, and a consistently larger proportion of downregulated than upregulated proteins (Fig. 6A; Fig. 6B). Functional enrichment analysis demonstrated a highly convergent biological signature: DEPs were mainly associated with lipid metabolic processes, particularly long-chain fatty acid metabolism, fatty acid transport, and fatty acid β-oxidation (Fig. 6C; Fig. 6D). KEGG analysis further confirmed that pathways involved in lipid metabolism—including fatty acid degradation, fatty acid metabolism, and the PPAR signaling pathway—were significantly enriched in both trials. In addition, amino acid metabolic pathways, such as arginine and proline metabolism and alanine, aspartate, and glutamate metabolism, were also affected, indicating broader metabolic disruptions beyond lipid catabolism (Fig. 6E; Fig. 6F). Reactome enrichment consistently highlighted alterations in lipid metabolism and general metabolic processes, supporting the notion of PEDV-induced metabolic reprogramming at the protein level (Fig. 6G; Fig. 6H). PPI network construction for each trial identified 20 hub proteins, further emphasizing key regulatory nodes involved in lipid metabolic pathways (Fig. S4; S5). Collectively, the proteomic data from trials 4 and 5 demonstrate a coherent and robust shift toward impaired lipid and amino acid metabolism following PEDV infection.

### Integrated analyses of transcriptomic and proteomic data

**Fig 7:**
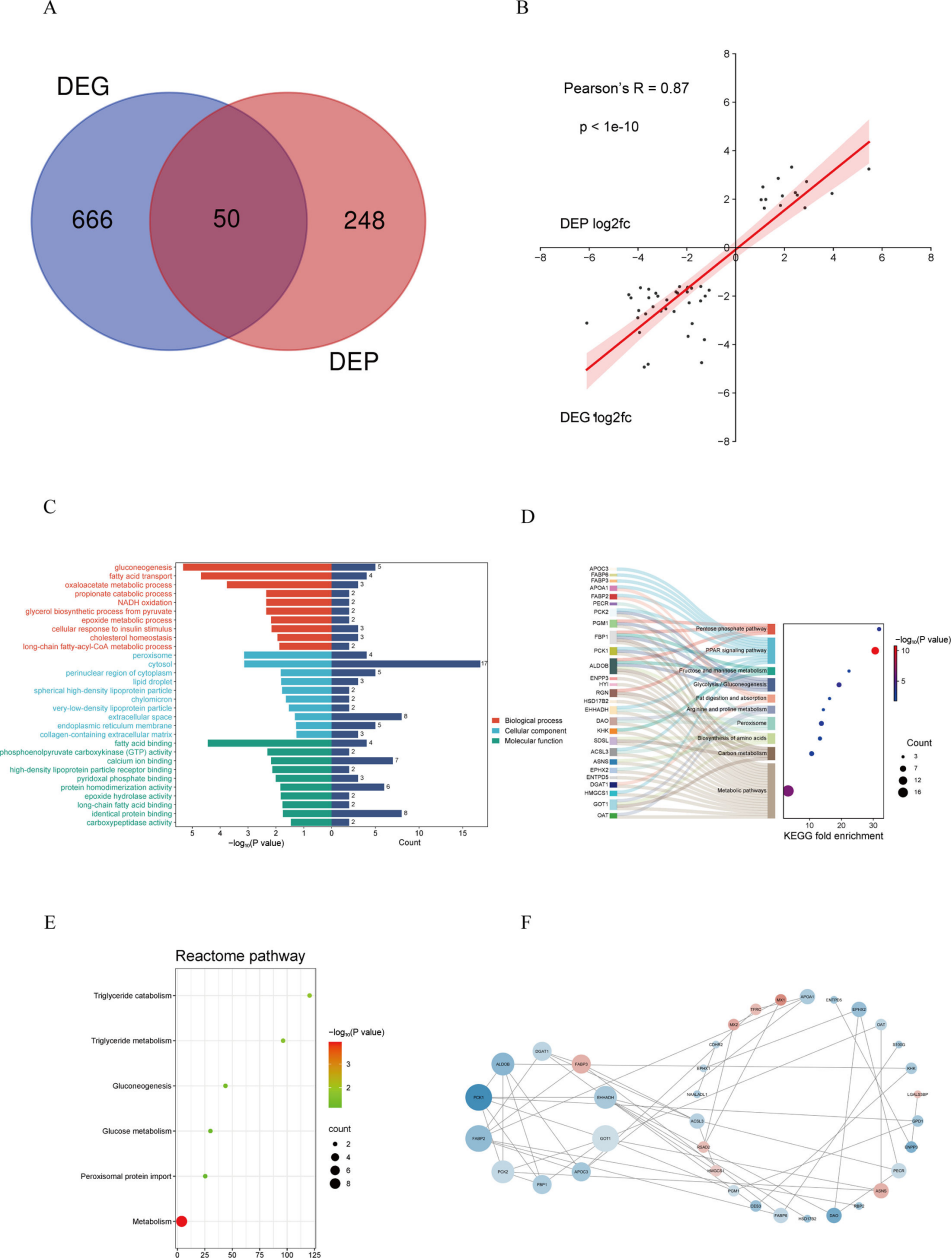
Integrated analysis of transcriptomic and proteomic data revealed co-regulated gene/protein networking after PEDV infection. (**A**) Venn diagram of differentially expressed genes (DEG) and proteins (DEP). (**B**) Correlation analysis between DEG and DEP. (**C**) GO analysis of overlapped DEG and DEP. (**D**) KEGG analysis of overlapped DEG and DEP. (**E**) Reactome analysis of overlapped DEG and DEP. (**F**) Protein-protein interaction analysis; the color intensity (red and blue) indicates fold change of the upregulated or downregulated genes/proteins, while the size of the circle suggests the degree of the interaction.

According to the Venn analysis, 50 DEPs from the comparison of the control and PEDV groups matched their transcripts (Fig. 7A). A correlation analysis was performed to investigate the congruence between transcriptomics and proteomics. Overall, the correlation coefficient between DEGs and DEPs was 0.87 (*P* < 1e-10)(Fig. 7B). GO analysis revealed that fatty acid transport and glycerol biosynthetic process were significantly enriched (Fig. 7C). Specifically, pathways related to fatty acid metabolism (particularly the PPAR signaling pathway) and amino acid metabolism (especially arginine and proline metabolism) were significantly enriched, as indicated by the Sankey diagram (Fig. 7D). Reactome analysis showed that metabolism, including triglyceride metabolism and triglyceride catabolism, was significantly enriched (Fig. 7E). In addition, 10 core genes/proteins were identified as the hub genes/proteins according to the PPI network (Fig. 7F).

### Proposed core pathway and hub genes influenced by PEDV in the small intestine of piglets

**Fig 8:**
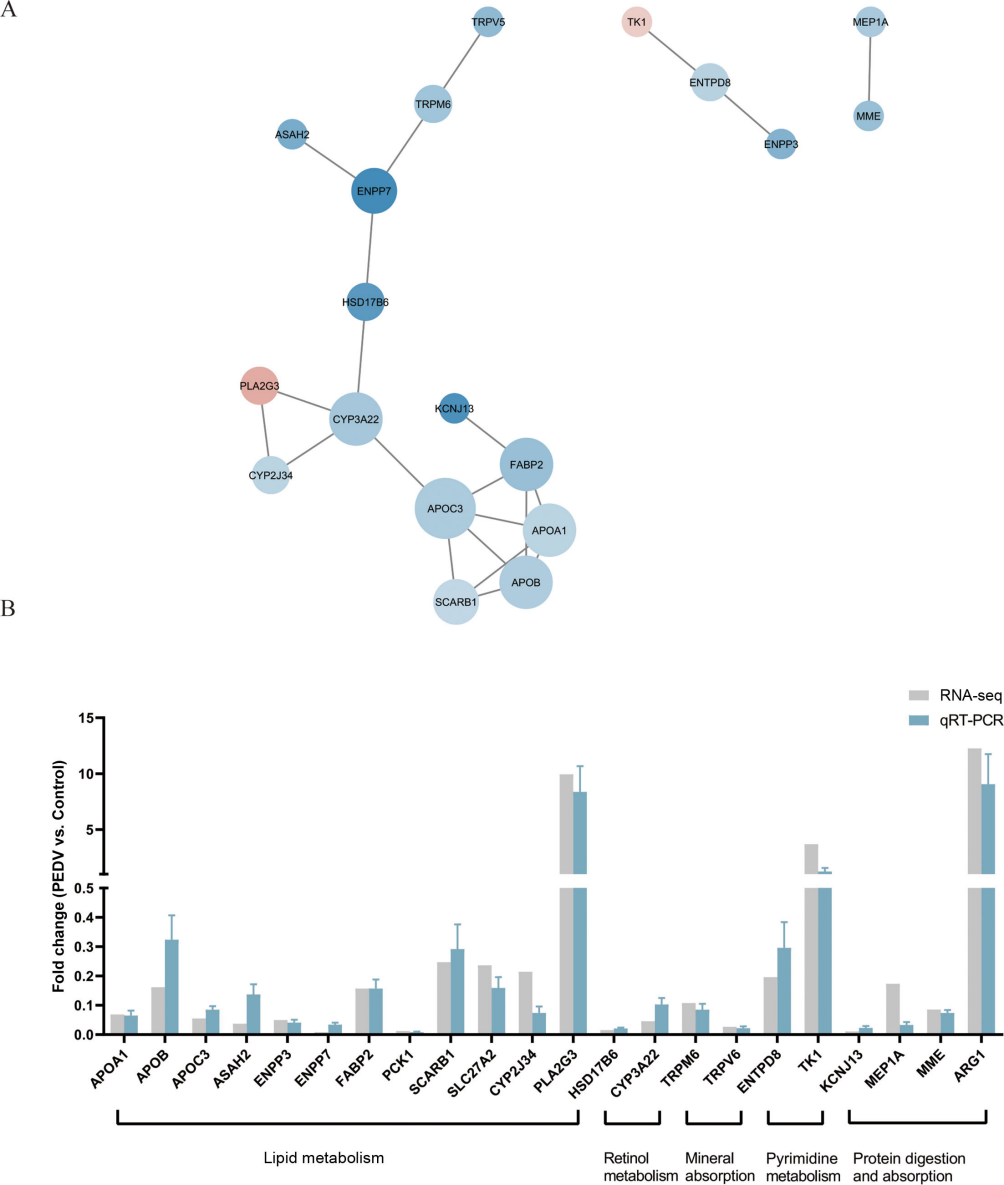
Validation of selected candidate hub genes. (**A**) Protein-protein interaction analysis of selected candidate hub genes. (**B**) Validation of hub genes by quantitative real-time PCR (qRT-PCR, *n* = 6, mean ± standard deviation).

After comprehensively reviewing the results from transcriptomic and proteomic data, the overlapping significant terms that appeared in each trial were considered the core pathways. Table 2 indicates that 16 pathways, including the IL-17 signaling pathway and cytokine-cytokine receptor interaction, were consistently upregulated, while 10 pathways, including mineral absorption, retinol metabolism, and sphingolipid metabolism, were consistently downregulated in PEDV-infected piglets. Table 3 suggests that 19 pathways, including metabolic pathways, protein digestion and absorption, fat digestion and absorption, PPAR signaling pathway, and retinol metabolism, were significantly enriched in KEGG pathway analysis, while 9 pathways, including metabolism, transport of small molecules, and metabolism of lipids, were significantly enriched in Reactome pathway analysis in PEDV-infected piglets. Given that KEGG and Reactome emphasize different aspects of biological processes, presenting both analyses allows us to capture complementary pathway information and provides a more comprehensive understanding of cellular responses to PEDV infection. The candidate hub genes were identified with three steps. Firstly, the overlapped significant terms that appeared in each trial were considered the core pathways, based on transcriptomic and proteomic data. Secondly, protein-protein interaction (PPI) was constructed using STRING combined with Cytoscape, based on the DEGs and DEPs results. We applied the cytoHubba plugin and ranked core genes based on degree-based network topology parameters. Finally, only genes that were both in the overlapping pathway and the PPI network were selected and considered as hub genes. Table 4 summarizes the core pathways and hub genes influenced by PEDV infection in the small intestine of piglets. The hub genes were validated by RT-qPCR and further analyzed using Cytoscape to construct a PPI network (Fig. 8).

**TABLE 2:** Significantly regulated pathways influenced by PEDV in the small intestine of piglets by gene set enrichment analysis (GSEA)

Alteration	GSEA (KEGG pathways)
Upregulated	IL-17 signaling pathway
	Cytokine-cytokine receptor interaction
	Fanconi anemia pathway
	Viral protein interaction with cytokine and cytokine receptor
	Cell cycle
	Rheumatoid arthritis
	DNA replication
	Nucleocytoplasmic transport
	Homologous recombination
	Mismatch repair
	Proteasome
	Cytosolic DNA-sensing pathway
	Spliceosome
	Ribosome biogenesis in eukaryotes
	Toll-like receptor signaling pathway
	
Downregulated	Sphingolipid metabolism
	ABC transporters
	Renin secretion
	Starch and sucrose metabolism
	Carbohydrate digestion and absorption
	Vitamin digestion and absorption
	Protein digestion and absorption
	Steroid hormone biosynthesis
	Mineral absorption
	Retinol metabolism

**TABLE 3:** Significantly regulated pathways influenced by PEDV in the small intestine of piglets by KEGG and Reactome enrichment analysis

Category	Pathways
KEGG	Metabolic pathways
	Protein digestion and absorption
	Fat digestion and absorption
	PPAR signaling pathway
	Retinol metabolism
	Cytokine-cytokine receptor interaction
	Pyrimidine metabolism
	Cholesterol metabolism
	Mineral absorption
	Vitamin digestion and absorption
	IL-17 signaling pathway
	Glycine, serine, and threonine metabolism
	Arachidonic acid metabolism
	Starch and sucrose metabolism
	Drug metabolism – other enzymes
	Carbohydrate digestion and absorption
	Bile secretion
	Biosynthesis of amino acids
	Linoleic acid metabolism
	
Reactome	Metabolism
	Transport of small molecules
	Metabolism of lipids
	Metabolism of nucleotides
	Biological oxidations
	Visual phototransduction
	Metabolism of amino acids and derivatives
	SLC-mediated transmembrane transport
	Metabolism of vitamins and cofactors

**TABLE 4:** Proposed core pathways and hub genes influenced by PEDV in the small intestine of piglets^*a*^

Category	Pathways	Genes
KEGG	Metabolic pathways	CYP3A22/CYP2J34/ENPP7
	Fat digestion and absorption	APOA1/APOB/SCARB1
	PPAR signaling pathway	APOA1/APOC3/FABP2/SLC27A2/PCK1
	Cholesterol metabolism	APOA1/APOB/APOC3/SCARB1
	Retinol metabolism	CYP3A22/HSD17B6
	Mineral absorption	TRPV6/TRPM6
	Protein digestion and absorption	MME/MEP1A/KCNJ13
	Arginine and proline metabolism	ARG1/ARG2
	Pyrimidine metabolism	ENTPD8/NME1/TK1
		
Reactome	Metabolism	CYP2J34/ENTPD8
	Metabolism of lipids	ENPP7/PLA2G3/ASAH2/FABP2
	Retinoid metabolism and transport	APOB/APOC3
	Transport of small molecules	APOB/APOC3/TRPV6/TRPM6
	Metabolism of nucleotides	ENTPD8/TK1

^*a*^ APOA1, apolipoprotein A1; APOB, apolipoprotein B; APOC3, apolipoprotein C3; ARG1, arginase 1; ARG2, arginase 2; ASAH2, N-acylsphingosine amidohydrolase 2; CYP3A22, cytochrome P450 family 3 subfamily A member 22; ENPP7, ectonucleotide pyrophosphatase/phosphodiesterase 7; FABP2, fatty acid-binding protein 2; HSD17B6, hydroxysteroid 17-beta dehydrogenase 6; KCNJ13, potassium inwardly rectifying channel subfamily J member 13; MEP1A, meprin A subunit alpha; MME, membrane metalloendopeptidase; PCK1, phosphoenolpyruvate carboxykinase 1; PLA2G3, phospholipase A2 group III; SCARB1, scavenger receptor class B member 1; SLC27A2, solute carrier family 27 member 2; TRPM6, transient receptor potential cation channel subfamily M member 6; TRPV6, transient receptor potential cation channel subfamily V member 6; ENPP3, ectonucleotide pyrophosphatase/phosphodiesterase 3; CYP2J34, cytochrome P450 family 2 subfamily J member 34; ENTPD8, ectonucleoside triphosphate diphosphohydrolase 8; TK1, thymidine kinase 1.

### Metabolomic analysis of blood in PEDV-infected piglets

**Fig 9:**
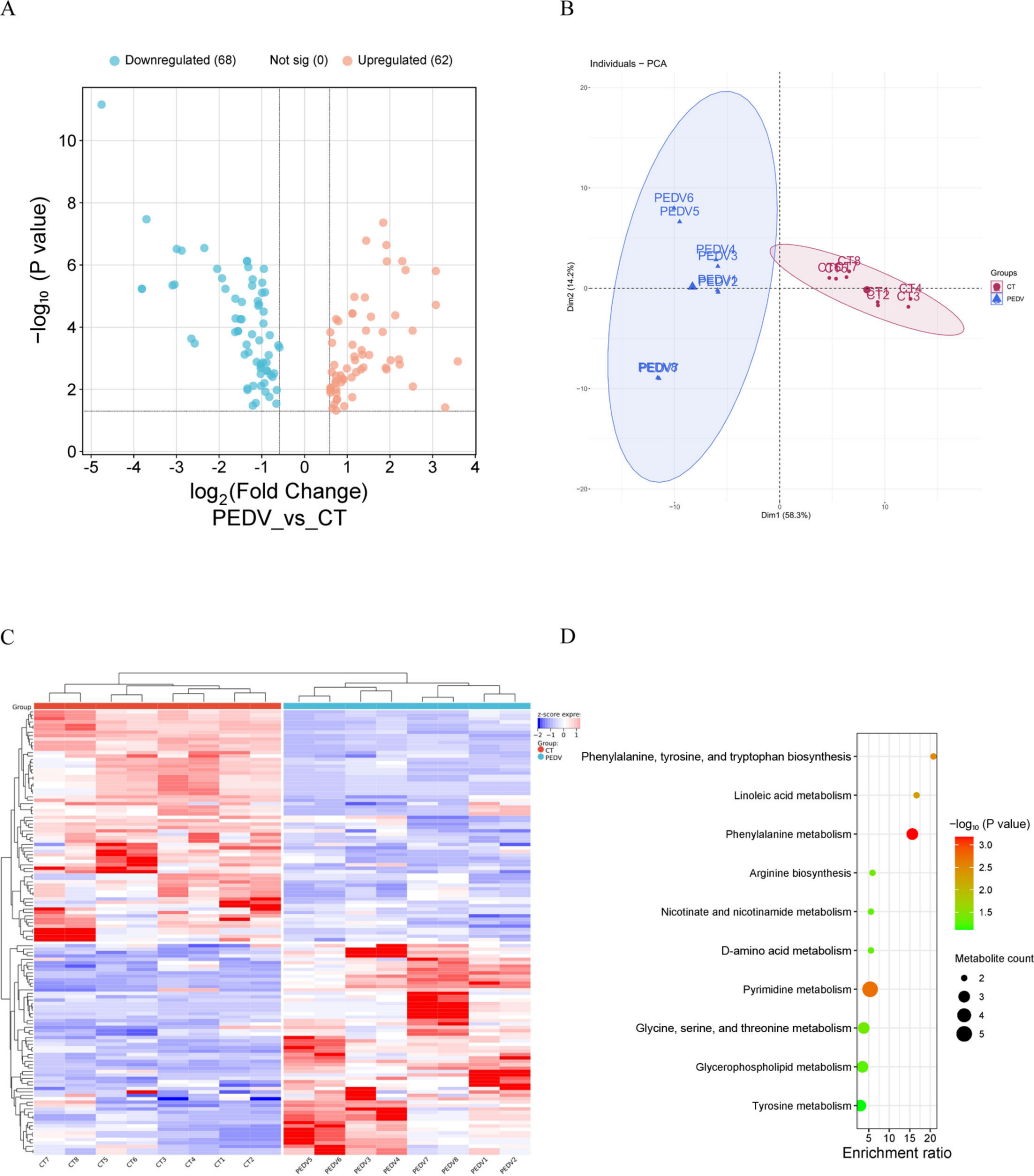
Metabolomic analysis of blood in PEDV-infected piglets from trial 7. (**A**) Volcano plot. (**B**) Principal component analysis (PCA). (**C**) Heatmap analysis. (**D**) KEGG analysis.

To validate the findings from the integrated analysis of transcriptomics and proteomics, an untargeted blood metabolomics analysis was therefore conducted. Compared with non-challenged piglets, 130 differentially expressed metabolites (DEMs) (62 upregulated and 68 downregulated) were identified in the PEDV group (Fig. 9A). Blood lysophosphatidylcholine, lysophosphatidylethanolamine, bile acids, coenzyme and vitamins, and amino acids were significantly decreased, while nucleotides and their metabolites, as well as organic acids and their derivatives, were increased after PEDV infection. Principal component analysis (PCA) indicated satisfactory reproducibility within groups and clear specificity between groups (PC1 = 58.3%, PC2 = 14.2%, Fig. 9B). The clustering heatmap indicated that DEM samples selected from each group exhibited similar expression pattern (Fig. 9C). KEGG analysis showed that lipid metabolism (including linoleic acid metabolism and glycerophospholipid metabolism) and amino acid metabolism (including phenylalanine metabolism and arginine biosynthesis) were significantly enriched (Fig. 9D).

### The effect of PEDV on plasma biochemical indicators in piglets

**Fig 10:**
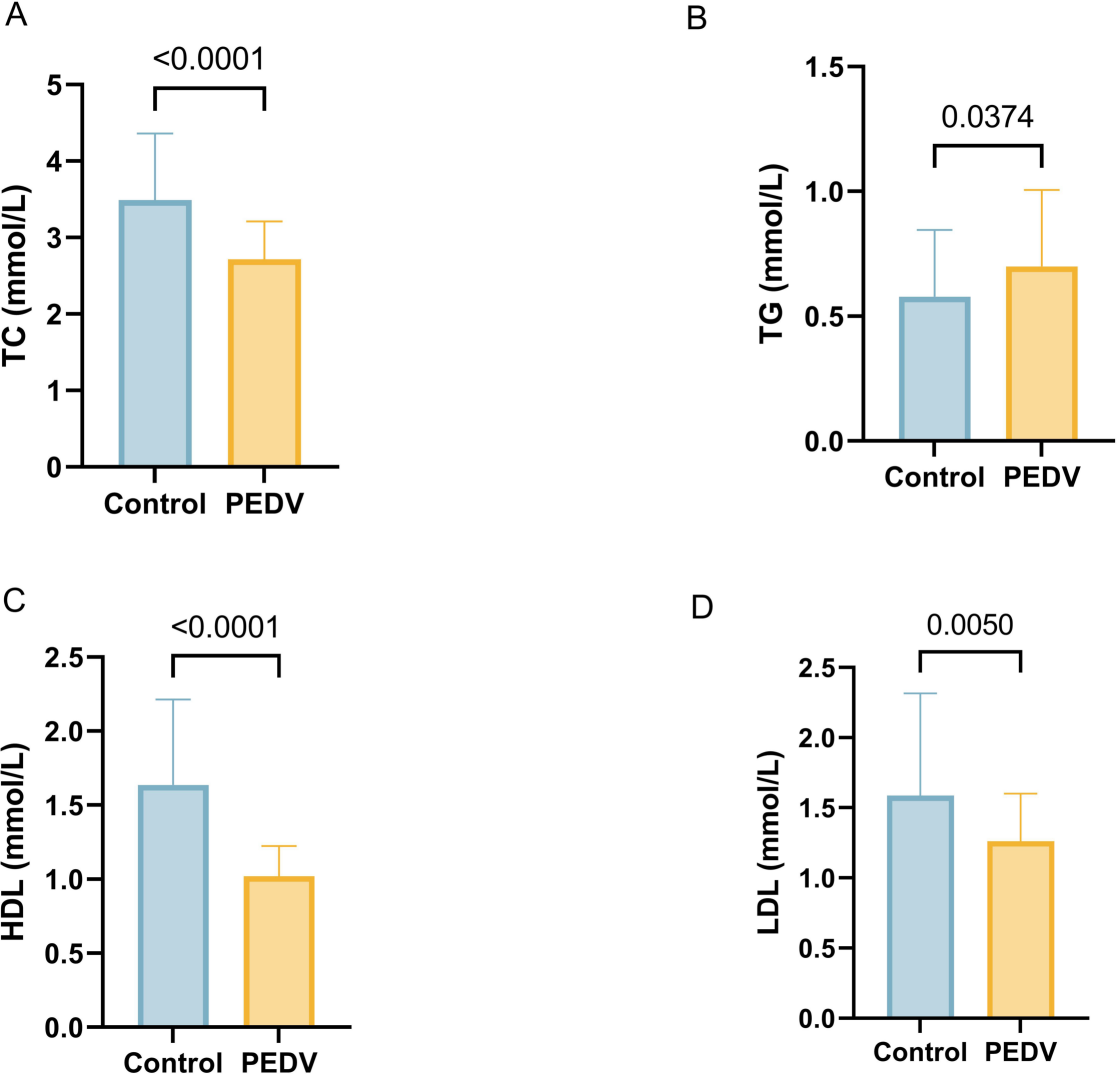
Effects of PEDV on plasma biochemical indicators in piglets. (**A**) Total cholesterol (TC). (**B**) Triglyceride (TG). (**C**) High-density lipoprotein (HDL). (**D**) Low-density lipoprotein (LDL). *n* = 50.

As shown in Fig. 10, PEDV infection significantly increased levels of triglycerides (TG) while decreasing levels of total cholesterol (TC), high-density lipoprotein (HDL), and low-density lipoprotein (LDL) in plasma (*P*＜0.05).

## DISCUSSION

Deciphering the regulatory networks underlying host responses to PEDV is crucial for identifying or developing therapeutic targets for PEDV infection (19). Based on a stable PEDV-induced intestinal injury model established earlier, we conducted seven independent animal trials at different times. The results of the present study demonstrate that the PEDV infection caused damage to intestinal morphology and resulted in diarrhea and reduced growth performance, which were consistent with previous studies (5, 6, 32). These findings indicate the significant destructive effect of PEDV on the intestinal cells. Transcriptomics, proteomics, and metabolomics have been widely adopted to identify functional genes, proteins, and metabolites involved in PEDV infection (8, 14, 16). However, the lack of integrative analysis from multi-omics data could influence the accuracy and robustness of predictive models (33). Furthermore, batch effects presented serious challenges for processing and combining data from different batches (34). In this study, we systematically demonstrated the dynamic profiles of transcriptomic, proteomic, and metabolomic alterations in PEDV-infected piglets via integrated omics and multi-batch analysis, revealing the intricate regulation of intestinal metabolism during PEDV infection. This pilot study was considered a discovery-driven multi-omics investigation rather than a comprehensive functional validation study. Nevertheless, these observations suggest that changes in the expression levels of these metabolites and proteins may serve as crucial regulating molecules and biomarkers for host cells infected by PEDV. The proposed therapeutic targets provided new insights into the prevention and control of PEDV.

### PEDV significantly affects intestinal cell metabolism

**Fig 11:**
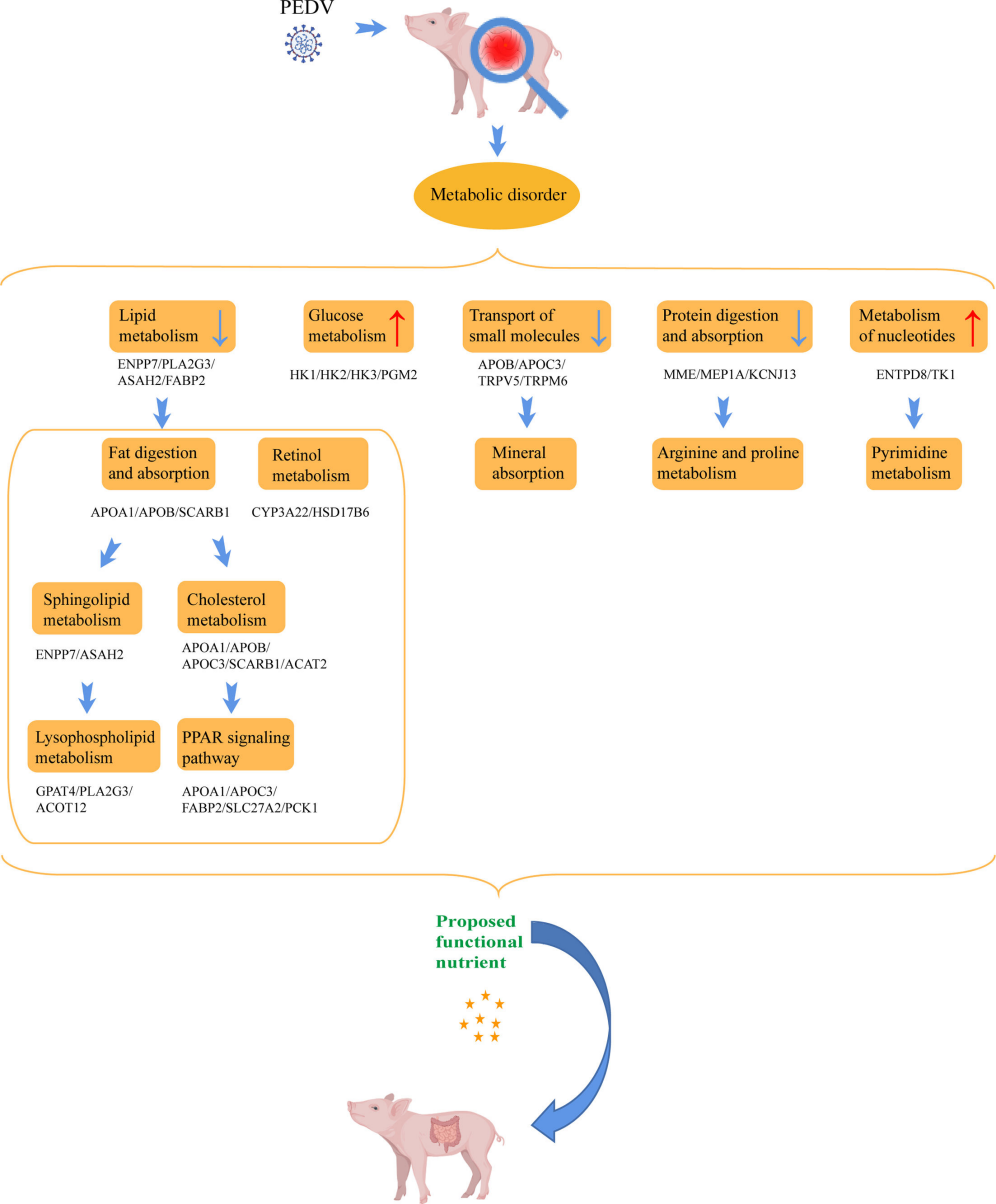
Schematic overview of the proposed model depicting how PEDV infection regulates host metabolism and potential therapeutic targets.

Viruses are obligate intracellular parasitic microorganisms, and interfering with the metabolic processes of host cells is one of the important procedures for viruses to promote replication or evade the host’s immune response (35). KEGG and Reactome pathway enrichment analyses consistently found significant changes in many metabolic pathways in PEDV-infected intestinal cells, including lipid and amino acid metabolism, which align with previous studies (6, 11, 32). In addition, retinol metabolism, mineral absorption, and pyrimidine metabolism were identified and validated as significantly influenced pathways during PEDV infection (Fig. 11). For instance, transcriptomic and proteomic analyses revealed that CYP3A22 and CYP2J34 were significantly downregulated following PEDV infection. The cytochrome P450 (CYP) system plays a crucial role in the metabolism of endogenous substances, such as steroids, fatty acids, prostaglandin, and cholesterol (36, 37). CYP2J34 is highly expressed in the small intestine of pigs (38), and CYP2J plays roles in the metabolism of endogenous substrates, such as arachidonic acid, thereby generating epoxyeicosatrienoic acids that are important for their protective roles in inflammation and vasodilation (39). A previous study found that influenza A treatment in mice resulted in a significant decrease in the expression of the CYP2J subfamily (40). In another study, CYP2J2 was shown to attenuate metabolic dysfunction in diabetic mice by reducing hepatic inflammation via the PPARγ (41). CYP3A is likely the primary CYP enzyme responsible for the metabolism of all-trans-retinoic acid in enterocytes (42). Retinoic acid plays a role in immune signal transduction against transmissible gastroenteritis virus and acts as an agonist of the nuclear receptor family, participating in lipid metabolism by regulating the PPAR signaling pathway (43). Clinical research suggested that the activity of CYP3A was significantly inhibited after viral infection (44). Thus, PEDV likely affects intestinal cell metabolism, including lipid metabolism or retinol metabolism, by inhibiting the expression of CYP3A22 and CYP2J34 in intestinal cells. This understanding helps elucidate how PEDV regulates host metabolism to promote self-replication and exacerbate the severity of diarrhea. Thus, studying the mechanisms by which PEDV regulates intestinal cell metabolism and designing therapeutic drugs or nutritional interventions targeting altered metabolic pathways have become an important antiviral strategy.

### PEDV significantly affects intestinal lipid metabolism

In this study, KEGG and Reactome analyses revealed that fat digestion and absorption in the intestine were significantly affected following PEDV infection. The increased concentration of TG in the blood confirmed that fat digestion and absorption were indeed affected by PEDV infection. Metabolomic analysis also revealed inhibition of bile acid secretion, with cholesterol being the only precursor molecule involved. The reduction in blood cholesterol suggests that the viral infection may have limited the liver’s ability to synthesize bile. Further, PPI analysis indicated that the expression levels of APOB, APOC3, APOA1, and SCARB1, molecules closely related to cholesterol metabolism, were significantly downregulated. Cholesterol lipoprotein metabolism relies on apolipoproteins, which are multifunctional proteins that serve as templates for lipoprotein particle assembly, maintain their structure, and direct their metabolism by binding to membrane receptors and regulating enzymatic activity (45). As the main component of HDL, APOA1 helps reduce cholesterol levels in the blood through reverse cholesterol transport and possesses multiple therapeutic functions (46). APOB is the structural protein of all non-HDL lipoproteins and mainly regulates cholesterol transport in LDL and VLDL (47). APOC3 mainly regulates triglyceride metabolism and indirectly affects cholesterol metabolism (48). SCARB1 is a scavenger receptor and an important mediator of HDL clearance and cholesterol transport to the liver during reverse cholesterol transport. In addition, SCARB1 is also related to bile acid synthesis (49). The reduction in LDL and HDL levels in the blood further indicates that cholesterol metabolism is affected. Previous studies have also reported that the expression of intestinal APOA1, APOA4, and APOC2 is downregulated in the jejunum and ileum of piglets after PEDV infection, which is consistent with our findings. The accumulation of cholesterol may also damage intestinal cells (32). Recent quantitative proteomics based on TMT confirmed that five DEPs in the cholesterol metabolism pathway were significantly downregulated after PK15 cells were infected with the PEDV wild strain and found that APOA1 may also be related to anti-inflammatory properties (14). In summary, this study shows that PEDV infection causes disorders in cholesterol metabolism and reduces bile secretion, leading to obstruction of lipid digestion and absorption. In addition, viral attachment to host cell receptors is lipid-dependent. Although the receptor for PEDV has not yet been identified, the receptor ACE2 of SARS-CoV-2, a coronavirus of the same family, is located in cholesterol-rich microdomains within lipid rafts (50). Lipid rafts are regions of the plasma membrane characterized by high concentrations of sphingolipids and cholesterol. Viral internalization occurs through endocytosis-mediated fusion of envelope lipids with the plasma membrane and cholesterol accumulation in the cell facilitates viral invasion. Therefore, cholesterol‐lowering agents can inhibit the optimal lipid microenvironment required for viral infection. On one hand, statins inhibit HMGR enzyme, the rate‐limiting enzyme in cholesterol biosynthesis, thereby reducing available cholesterol and blocking viral entry (51, 52). Plant phytosterols, such as betulinic acid, are lipophilic compounds with cholesterol‐like structures that can interact with lipid rafts, decrease membrane cholesterol, and thus inhibit viral attachment to host cells (53, 54). On the other hand, promoting the metabolism and transfer of cholesterol by increasing the expression of APOA1, APOB, APOC3, and SCARB1 could also be a potential strategy to reduce cholesterol concentration in cells. Moreover, in the current study, intestinal acyl-CoA cholesterol acyltransferase 2 (ACAT2) transcript levels were significantly increased after PEDV infection. One study found that acyl-CoA cholesterol acyltransferase plays an important role in mediating lipid raft and lipid droplet formation (50, 55). Recent studies report that pharmacological inhibition of ACAT reduces hepatitis B and C virus replication (56, 57). This indicates that it may be possible to inhibit PEDV replication by limiting the formation of lipid raft and lipid droplet through interference with ACAT2 expression.

Cholesterol synthesis is derived from acetyl-CoA, which can be produced through fatty acid β-oxidation and glycolysis. In the present study, we found that fatty acid β-oxidation was inhibited after PEDV infection, while glycolysis was activated to provide pyruvate, a precursor for acetyl-CoA production. However, gluconeogenesis was suppressed by PEDV infection. This finding aligns with a previous study that indicated PEDV may rig metabolic processes in which pyruvate acts as a key metabolite to promote its replication in host cells (6). Furthermore, cholesterol metabolism is regulated by the peroxisome proliferator-activated receptor (PPAR) signaling pathway, a family of transcription factors (58). In this study, we found that the PPAR pathway (including genes such as APOA1, FABP2, SLC27A2, and PCK1) was significantly inhibited by PEDV. PPARα activation can increase APOA1 levels and participate in cholesterol metabolism and lipid transport (59). FABP2 is mainly expressed in the distal part of the small intestine and plays an important role in the absorption, transport, and metabolism of fatty acids, thereby helping to maintain energy metabolism balance (60). SLC27A2 (long-chain fatty acid transporter 2) is a major fatty acid transporter in intestinal cells responsible for transporting long-chain fatty acids into cells across the plasma membrane, enhancing fatty acid β-oxidation and providing energy through the regulation of PPARα and PPARγ (61). Phosphoenolpyruvate carboxykinase 1 (PCK1) is the first rate-limiting enzyme in gluconeogenesis. PCK1 not only regulates glucose homeostasis but also influences lipogenesis by activating sterol regulatory element-binding proteins through the PPAR signaling pathway or promoting fatty acid degradation (62). Fatty acid β-oxidation is a key step in fatty acid catabolism, and its regulation to promote viral self-replication has been widely reported. SARS-CoV-2, hepatitis C virus, Japanese encephalitis virus, and influenza A virus downregulate β-oxidation, thereby providing cytoplasmic free fatty acids for viral replication (35). It has been observed that PEDV may inhibit long-chain fatty acid β-oxidation by reducing the expression of long-chain acyl-CoA dehydrogenase (25). This is consistent with our results. In addition, a recent study revealed that PEDV infection can block the cell cycle and induce apoptosis of porcine intestinal epithelial cells by disrupting energy metabolism (9). Thus, we speculate that the degradation of fatty acids inhibited by PEDV may lead to an insufficient intestinal energy supply in the intestines, thereby hindering the growth and renewal of intestinal epithelial cells.

Furthermore, metabolomic results indicated that PEDV infection reduced levels of lysophosphatidylcholine (LPC) and lysophosphatidylethanolamine (LPE) in the blood. Similarly, previous studies found that the four LPC metabolites were at their lowest levels in PEDV-infected pigs (11). A study combining metabolomic and lipidomic serum analysis of clinical pigs with *in vivo* and *in vitro* infection models, utilizing machine learning methods, found that LPC and LPE were reduced following PRRS virus (PRRSV) infection (19). Coronaviruses can manipulate host cell pathways, particularly those related to lipid metabolism and lipid transport, to form replication and transcription complexes, namely double-membrane vesicles (DMVs), thereby providing a favorable barrier to protect the viral replication compartment from the host’s innate immune response. The rapid biogenesis of DMV requires lysophospholipids (LPLs) (50). In addition, we found that the expression of PLA2G3 was rapidly increased after PEDV infection. Cytosolic PLA2α (cPLA2α) is a critical lipid-processing enzyme that plays a key role in the formation of DMVs (63). The reduction of LPL in the blood following PEDV infection indicates that LPL may be processed by PLA2G3 in intestinal cells for DMV synthesis necessary for viral replication. Similarly, to identify the cellular lipids critical for West Nile virus strain Kunjin virus replication, a previous study utilized a whole-cell lipidomics approach and revealed that the PLA2 enzyme family is activated in West Nile virus strain Kunjin virus-infected cells to produce lysophosphatidylcholine (64). Therefore, reducing LPL synthesis by inhibiting PLA2G3, resulting in the loss of DMVs, could be a potential strategy to inhibit coronavirus replication. A previous study found that cPLA2α inhibitors, such as pyrrolidine‐2 or AM580 (a stable retinobenzoic acid derivative), significantly decreased DMV formation (65, 66). Polyunsaturated fatty acids (PUFAs) play important roles in regulating cPLA2α and DMV formation; therefore, exogenous supplementation of PUFAs can suppress viral infection via the negative feedback inhibition mechanism on cPLA2α (67). It has been shown that PUFAs can inactivate SARS‐CoV‐2 by blocking viral proliferation and inducing leakage and lysis of the viral envelope (68). Consequently, PUFA supplementation could reduce susceptibility to PEDV infection.

**Fig 12:**
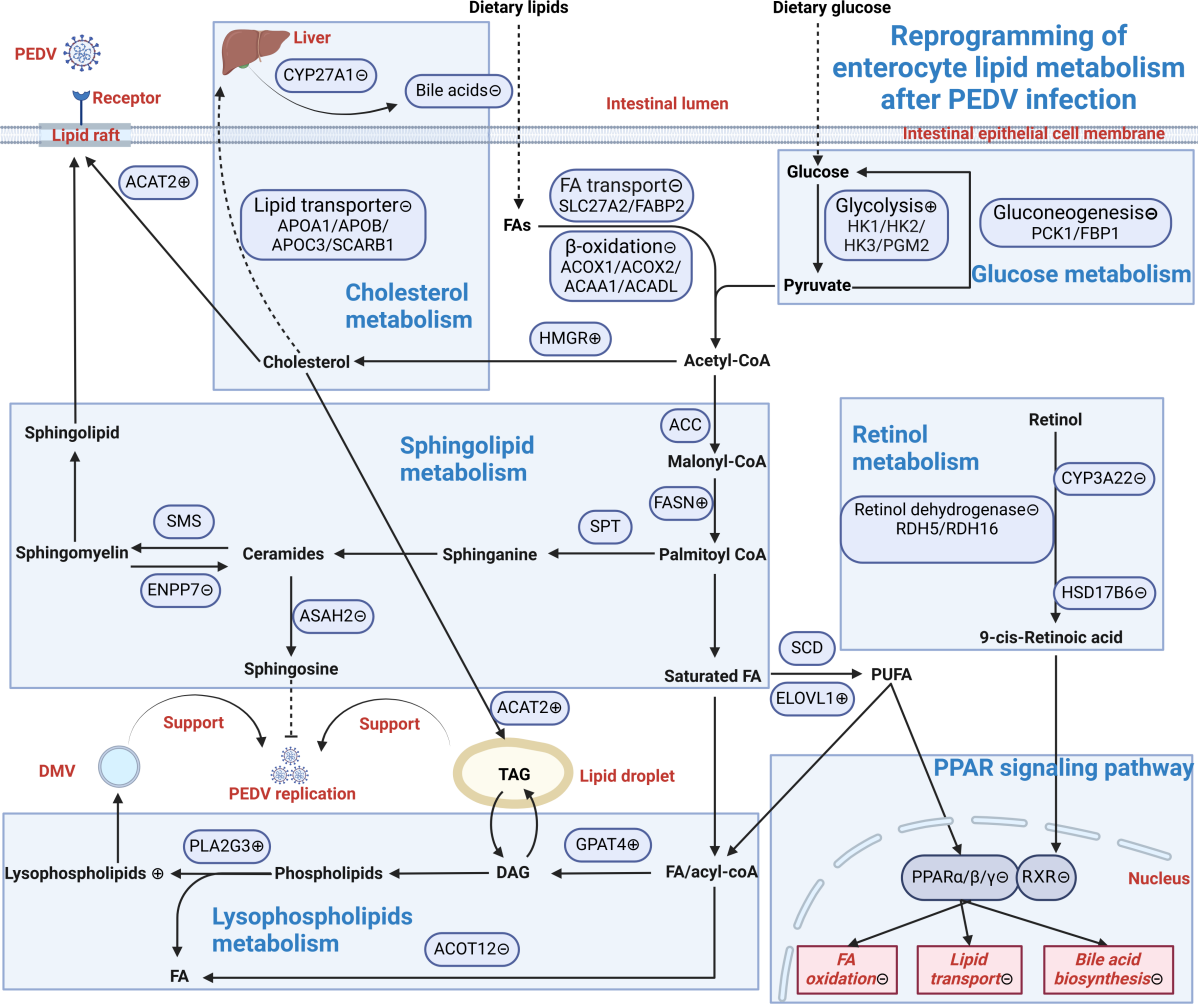
Proposed role of PEDV in the lipid metabolism of intestinal epithelial cells. ACAA1, acetyl-CoA acyltransferase 1; ACADL, long-chain specific acyl-CoA dehydrogenase; ACAT2, acyl-CoA cholesterol acyltransferase 2; ACC, acetyl-CoA carboxylase; ACOT12, acetyl-coenzyme A thioesterase; ACOX1, acyl-CoA oxidase 1; ACOX2, acyl-CoA oxidase 2; APOA1, apolipoprotein A1; APOB, apolipoprotein B; APOC3, apolipoprotein C3; ASAH2, N-acylsphingosine amidohydrolase 2; CYP27A1, cytochrome P450 family 27 subfamily A member 1; CYP3A22, cytochrome P450 family 3 subfamily A member 22; DAG, diacylglycerol; DMV, double-membrane vesicles; ELOVL1, ELOVL fatty acid elongase 1; ENPP7, ectonucleotide pyrophosphatase/phosphodiesterase 7; FABP2, fatty acid-binding protein 2; FAs, fatty acids; FASN, fatty acid synthase; FBP1, fructose-1,6-bisphosphatase; GPAT4, glycerol-3-phosphate acyltransferase 4; HK1, hexokinase 1; HK2, hexokinase 2; HK3, hexokinase 3; HMGR, 3-hydroxy-3-methylglutaryl-coenzyme A reductase; HSD17B6, hydroxysteroid 17-beta dehydrogenase 6; PCK1, phosphoenolpyruvate carboxykinase 1; PEDV, porcine epidemic diarrhea virus; PGM2, phosphoglucomutase 2; PLA2G3, phospholipase A2 group III; PPAR, peroxisome proliferator-activated receptor; RDH16, retinol dehydrogenase 16; RDH5, retinol dehydrogenase 5; RXR, retinoid X receptor; SCARB1, scavenger receptor class B member 1; SCD, stearoyl-CoA desaturase; SLC27A2, solute carrier family 27 member 2; SMS, sphingomyelin synthase; SPT, serine palmitoyltransferase; TAG, triacylglycerol.

It is worth noting that PEDV also significantly downregulated the sphingolipid metabolic pathway. Recent advances in multi-omics analysis have shown that human health and disease are closely related to the homeostasis of sphingolipid metabolism (69). In this study, we first discovered that intestinal alkaline sphingomyelinase ENPP7 and neutral ceramidase N-acylsphingosine amide hydrolase 2 (ASAH2) were significantly inhibited by PEDV. Sphingolipids are degraded in the intestine by sphingomyelinase and ceramidase into ceramide and sphingosine (70), which can prevent SARS-CoV-2 infection by interfering with the interaction of the virus with its receptor (71) and may also inhibit influenza A virus replication by disrupting the viral life cycle (72). Currently, a major challenge for sphingolipid metabolism is to decipher the specific biochemical regulation of sphingolipid metabolic enzymes and their products (73). Recent research has found that the metabolite sphingosine, secreted by *Enterobacter horsi*_B17, inhibits Zika virus in *Aedes aegypti* and cell cultures by blocking viral entry during the fusion step (74). Ceramides were found to significantly inhibit porcine rotavirus infection, exerting their antiviral effect during the replication phase of infection (75). PEDV infection is more likely to achieve immune evasion by inhibiting the metabolism of sphingolipids into ceramides with immunomodulatory effects and sphingosine with antiviral effects. Conversely, accumulated sphingolipids provide necessary lipid materials for the synthesis of lipid rafts, thereby promoting virus invasion and replication. Interestingly, a recent study demonstrated that *Bacillus subtilis* DE111 increases the expression of protein phosphodiesterase ENPP7 and ceramidase ASAH2, both of which are involved in fatty acid and lipid metabolism, suggesting that DE111 plays a beneficial role in digestive, metabolic, and immune health (76). However, the roles of ASAH2, ENPP7, as well as the above-mentioned APOC3, CYP3A22, and PLA2G3, remain inferential and require future functional validation. Given the functional role of sphingolipids in immunity and their significance in diseases, future research could explore the regulation of ENPP7 and ASAH2 to influence the sphingolipid metabolic pathway in alleviating PEDV infection. In addition, the proposed role of PEDV in intestinal epithelial cell lipid metabolism is summarized in Fig. 12.

### PEDV significantly affects intestinal retinol metabolism

Interestingly, we found that retinol metabolism was significantly inhibited after PEDV infection, which was similar to the pigs infected with ASFV (77). Retinol (also known as vitamin A) is a fat-soluble vitamin and an essential nutrient for animals, and its derivatives include retinaldehyde and retinoic acid (RA) (78, 79). RA has been confirmed to have various biological effects on the intestine, including enhancing immune function (79–81). The molecular mechanisms underlying the effects of retinoids on lipid metabolism are complex and not yet fully understood. Evidence suggests that specific retinoids affect mammalian adipogenesis, lipolysis, and fatty acid oxidation in tissues (43). Previous studies have found that CYP3A may be the main CYP enzyme responsible for all-trans retinoic acid metabolism in intestinal cells (42), while a recent study demonstrated that 17β-hydroxysteroid dehydrogenase type 6 (HSD17B6) is another retinol dehydrogenase (81). In this study, the expression of retinol metabolism-related genes CYP3A22, HSD17B6, RDH5, and RDH16 was significantly inhibited following PEDV infection. Recent studies have confirmed that changes in CYP3A22 and HSD17B6 expression affect steroid hormone biosynthesis and retinol metabolism pathways (82). RDH16 can inhibit cancer stem cell self-renewal through the synthesis of ATRA, which may present a potential target for glioma treatment (83), while RDH5 plays a vital role in inhibiting proliferation and metastasis, making it a potential therapeutic target for patients with hepatocellular carcinoma (84). Given the important roles of CYP3A22, HSD17B6, RDH5, and RDH16 in retinol metabolism, they are likely critical targets for improving intestinal lipid metabolism in PEDV-infected piglets. In addition, PPAR is a nuclear hormone receptor that can be activated by unsaturated fatty acids and their derivatives, including retinoids. The natural agonists of the PPAR pathway, such as eicosapentaenoic acid (EPA) (85) and all-trans-retinoic acid (ATRA), the natural ligand of the retinoic acid receptor (86), may be suitable candidates for alleviating intestinal lipid metabolism disorders caused by PEDV.

### PEDV significantly affects intestinal mineral absorption

Our findings indicate that “Transport of small molecules” was significantly enriched following PEDV infection. KEGG analysis revealed that, in addition to the above-mentioned abnormalities in lipid metabolism, intestinal mineral absorption also underwent significant changes, which is consistent with the previous research (6). Minerals, such as calcium, magnesium, and potassium, are essential nutrients vital for sustaining life (87, 88) (89). Minerals could be absorbed through the intestinal mucosa via passive or active transport mechanisms that utilize specialized transport proteins, such as transient receptor potential cation channel subfamily V member 6 (TRPV6, a calcium channel protein), transient receptor potential cation channel subfamily M member 6 (TRPM6, a magnesium channel protein), and potassium inwardly rectifying channel subfamily J member 13 (KCNJ13). Previous studies have shown that PEDV infection significantly decreases the activities of Na^+^–K^+^-ATPase and Ca^2+^–Mg^2+^-ATPase in enterocytes, inducing abnormal distributions of Na^+^, K^+^, Ca^2+^, and Mg^2+^ (32). Our study found that PEDV infection significantly reduced the relative expression of TRPV6 and KCNJ13 genes, corroborating earlier findings that both serum calcium levels and relative expression of TRPV6 and KCNJ13 in the small intestine decreased following PEDV infection (5, 90). A pilot study demonstrated that PEDV proliferation was inhibited by reducing the loss of PEDV-induced calcium channel protein (TRPV6), which helped restore the balance of intracellular and extracellular Ca2+ concentrations in porcine small intestinal epithelial cells (IPEC-J2) (91). In addition, we found for the first time that PEDV also inhibits the expression of TRPM6, whose deficiency is closely associated with diseases that impair intestinal absorption, such as inflammatory bowel disease and celiac disease (92). However, the mechanism by which PEDV downregulates TRPM6 remains unclear and will be a focus of our future research. In conclusion, our study found that PEDV infection impaired the expression of potassium, calcium, and magnesium channel protein genes, severely disrupting intestinal mineral homeostasis. Enhancing intestinal transport channels for potassium, calcium, and magnesium ions may represent a potential therapeutic strategy, providing a theoretical basis for the development of effective drugs or nutritional feed additives.

### PEDV significantly affects intestinal arginine and proline metabolism

A prominent feature of metabolic disorders in PEDV-infected intestinal cells is the impairment of protein digestion and absorption, particularly concerning the amino acid metabolism, in which arginine and proline metabolism are the most critical. Increasing evidence indicates a close link between viral infections and amino acid metabolism; for instance, coronaviruses, including SARS-CoV-2 and transmissible gastroenteritis virus (TGEV), interfere with arginine metabolism, suggesting that arginine may play a vital role in the coronavirus life cycle (93, 94). Arginine serves as a precursor for nitric oxide synthesis and is involved in various functions related to protein synthesis, immune response, and viral infection (95, 96). The present study found that PEDV infection significantly increased the relative expression of ARG1, which was consistent with previous research on African swine fever virus (ASFV) (97). Given that nitric oxide is a key mediator of innate inflammatory immune responses triggered by viral infections (98), it is worth exploring whether PEDV can affect nitric oxide production by modulating arginase levels, potentially creating favorable conditions for viral replication. Interestingly, we also found for the first time that PEDV infection significantly increased the relative expression of ARG2, which, like ARG1, catalyzes the conversion of arginine to ornithine, thereby providing substrates for proline synthesis and the polyamine pathway. A previous study has indicated that polyamines could promote PEDV replication in Vero cells (99). Therefore, inhibiting the expression of ARG1 and ARG2 to limit arginine and proline metabolism may represent a viable strategy to reduce PEDV replication and mitigate intestinal damage in piglets. More experiments should be scheduled in the future work, including siRNA/CRISPR-Cas9 manipulation in IPEC-J2 cells, overexpression assays, and pharmacological modulation of ACAT2, ARG1/2, ATRA, and PUFAs, to establish their proviral or host-protective functions.

### PEDV significantly affects intestinal pyrimidine metabolism

Viruses rely on the metabolic pathways of host cells to synthesize the nucleotides necessary for genome construction. Our study found that PEDV infection upregulated nucleotide metabolic pathways, particularly the pyrimidine metabolic pathway, which includes thymidine kinase 1 (TK1) and exonucleoside triphosphate diphosphate hydrolase 8 (ENTPD8). Metabolomic analyses revealed that blood nucleotides, such as thymidine and uridine and their metabolites, were significantly increased by PEDV infection. Consistent with this finding, recent studies have demonstrated that PEDV heavily depended on the host’s pyrimidine metabolism to produce a large amount of uridine for RNA genome replication (100, 101). TK1 is a kinase involved in pyrimidine salvage, phosphorylating thymidine to form TMP and playing an important role in regulating nucleotide metabolism (102). ENTPD8 is a key exonucleotidase involved in pyrimidine metabolism, capable of hydrolyzing nucleoside triphosphates and nucleoside diphosphates into their monophosphate forms (103). Inhibiting viral replication by blocking pyrimidine nucleotide synthesis has been proven to be feasible in a previous study on canine distemper virus (104). Recent studies have also shown that effective drugs have been successfully developed to inhibit PEDV *in vitro* by targeting pyrimidine metabolism and competing with uracil (105). In summary, our findings suggest that targeting pyrimidine metabolism by downregulating the expression of TK1 and ENTPD8 may provide an effective strategy for inhibiting PEDV.

### Conclusion

The findings of this study demonstrate that PEDV infection leads to metabolic reprogramming in enterocytes, particularly affecting lipid metabolism. Integrated multi-omics analyses indicate that PEDV infection induces extensive reprogramming of host lipid metabolism, consistent with the established strategy of coronaviruses to exploit host metabolic resources for viral envelope formation and efficient release. Furthermore, our study highlights the potential benefits of improving lipids metabolism, retinol metabolism, mineral absorption, amino acid metabolism, and pyrimidine metabolism in alleviating PEDV-induced intestinal injury in piglets. Candidate targets for preventive and therapeutic intervention include apolipoprotein C3 (APOC3), cytochrome P450 family 3 subfamily A member 22 (CYP3A22), and intestinal alkaline sphingomyelinase (ENPP7). Further studies are required to understand the biological impacts of the identified core pathways and differentially expressed genes, proteins, and metabolites (DEGs/DEPs/DEMs) during PEDV infection. Whether targeting these pathways and molecules confers therapeutic benefits against PEDV infection will be addressed in future studies.

## Data Availability

The RNA-seq data have been deposited in the NCBI Sequence Read Archive under the BioProject accession numbers PRJNA1428542, PRJNA1428930, and PRJNA1429256. The mass spectrometry proteomics datasets have been deposited to the ProteomeXchange Consortium (https://proteomecentral.proteomexchange.org) via the iProX partner repository (106, 107) with the identifier PXD075013 (Subproject ID IPX0015897001, IPX0015897002, and IPX0015897003). The metabolism data reported in this paper have been deposited in the OMIX, China National Center for Bioinformation/Beijing Institute of Genomics, Chinese Academy of Sciences, with the accession numbers OMIX015337 (https://ngdc.cncb.ac.cn/omix). The other data sets used and/or analyzed during the current study are available from the corresponding author on reasonable request.

## SUPPLEMENTAL MATERIAL

Supplemental material spectrum.01625-25-s0001.docx Supplemental results; Fig. S1 to S5.

ASM does not own the copyrights to Supplemental Material that may be linked to, or accessed through, an article. The authors have granted ASM a non-exclusive, world-wide license to publish the Supplemental Material files. Please contact the corresponding author directly for reuse.
