# Supplementary material for: Metabolic reprogramming in the enterocytes of neonatal piglets infected with porcine epidemic diarrhea virus: integrated omics and multi-batch analysis highlight alterations in lipid metabolism and potential therapeutic targets
Source: Microbiol Spectr. 2026 Apr 30;14(6):e01625-25. doi: 10.1128/spectrum.01625-25 (PMC13227961; doi:10.1128/spectrum.01625-25)
Supplement: Supplemental material — Supplemental results; Fig. S1 to S5. [file spectrum.01625-25-s0001.docx]

**Metabolic reprogramming in the enterocytes of neonatal piglets infected with porcine epidemic diarrhea virus: Integrated omics and multi-batch analysis highlight alterations in lipid metabolism and potential therapeutic targets**

Mengjun Wu^1,2,#^, Qian Zhang^1,2,#^, Xintao Shi^1,2,#^, Peng Li^1,2^, Zhuan Song^1,2^,

Zhonghua Li^1,2^, Yanyan Zhang^1,2^, Lei Wang^1,2^, Di Zhao^1,2^, Tao Wu^1,2^, Dan Yi^1,2^,

Yongqing Hou^1,2,*^

^1^Hubei Key Laboratory of Animal Nutrition and Feed Science, Wuhan Polytechnic University, Wuhan 430023, China;

^2^Engineering Research Center of Feed Protein Resources of Agricultural By-products, Ministry of Education, Wuhan Polytechnic University, Wuhan 430023, China;

^#^These authors share the first authorship

^*^Correspondence: [houyq@aliyun.com](mailto:houyq@aliyun.com)

Running head: PEDV REPROGRAM LIPID METABOLISM OF ENTEROCYTES

**SUPPLEMENTARY RESULTS**

**Transcriptomic analysis of the small intestine in PEDV-infected piglets**

Gene Set Enrichment Analysis (GSEA) is a threshold-free analysis method without prior gene filtering, which is particularly suitable and recommended for the global analysis of transcriptomic data. In trial 1, GSEA analysis showed that among the top 10 enriched KEGG pathways, 6 pathways, including cytokine-cytokine receptor interaction and IL-17 signaling pathway, were positive-enriched, while 4 pathways, such as retinol metabolism and mineral absorption, were negative-enriched (Figure 3A). In addition, compared with none-challenged piglets, 716 DEGs (329 up-regulated and 387 down-regulated) were identified in the PEDV group (Figure 3B). To understand the biological processes associated with PEDV pathogenesis, GO and KEGG analyses were applied to the aforementioned DEGs. The DEGs between the control and PEDV groups were primarily enriched in biological processes related to immune responses (Figure 3C). KEGG analysis showed that digestion and absorption (e.g., vitamin and fat), metabolism (e.g., cholesterol, retinol, pyrimidine metabolism) were significantly enriched (Figure 3D). Reactome analysis showed that retinoid metabolism and transport of small molecules were significantly enriched (Figure 3E). In addition, twenty core genes were identified as the hub genes according to the PPI network (Figure S1).

In trial 2, GSEA analysis indicated that among the top 10 enriched KEGG pathways, 7 pathways, including cytokine-cytokine receptor interaction and IL-17 signaling pathway, were positively enriched, while 3 pathways, such as retinol metabolism, vitamin digestion and absorption and mineral absorption, were negatively enriched (Figure 4A). In addition, compared with none-challenged piglets, 688 DEGs (316 up-regulated and 372 down-regulated) were identified in the PEDV group (Figure 4B). The DEGs between the control and PEDV groups were primarily enriched in biological processes including transmembrane transport, high-density lipoprotein particle remodeling, etc. (Figure 4C). KEGG analysis showed that digestion and absorption (e.g., vitamin and fat) and metabolism (e.g., cholesterol, retinol, pyrimidine metabolism) were significantly enriched (Figure 4D). Reactome analysis showed that retinoid metabolism and transport and transport of small molecules, etc. were significantly enriched (Figure 4E). In addition, twenty core genes were identified as the hub genes according to the PPI network (Figure S2).

In trial 3, GSEA analysis showed that among the top 10 enriched KEGG pathways, 6 pathways, including cell cycle, ribosome biogenesis in eukaryotes, and proteasome, etc., were positively enriched, while 4 pathways, such as retinol metabolism, steroid hormone biosynthesis, and mineral absorption, were negatively enriched (Figure 5A). In addition, compared to none-challenged piglets, 796 DEGs (357 up-regulated and 439 down-regulated) were identified in the PEDV group (Figure 5B). The DEGs between the control and PEDV groups were mainly enriched in biological processes of cholesterol homeostasis, retinol metabolic process, etc. (Figure 5C). KEGG analysis showed that digestion and absorption (e.g., vitamin and fat), metabolism (e.g., cholesterol, retinol, pyrimidine metabolism) were significantly enriched (Figure 5D). Reactome analysis showed that retinoid metabolism and transport and transport of small molecules, etc. were significantly enriched (Figure 5E). In addition, twenty core genes were identified as the hub genes according to the PPI network (Figure S3).

**Proteomic analysis of small intestine in PEDV-infected piglets**

In trial 4, a total of 177 DEPs (56 up-regulated and 121 down-regulated) were identified in the PEDV group (Figure 6A). The DEPs between the control and PEDV groups were primarily enriched in biological processes related to lipid metabolic process (e.g., long-chain fatty acid metabolic process, fatty acid transport, fatty acid beta-oxidation) (Figure 6C). KEGG analysis showed that lipid metabolism (fatty acid degradation, PPAR signaling pathway, fatty acid metabolism) and amino acid metabolism (arginine and proline metabolism, alanine, aspartate and glutamate metabolism) were significantly enriched (Figure 6E). Reactome analysis suggested that metabolism, metabolism of lipids, etc. were significantly enriched (Figure 6G). In addition, twenty core proteins were identified as hub proteins according to the PPI network (Figure S4).

In trial 5, 300 DEPs (101 up-regulated and 199 down-regulated) were identified in the PEDV group (Figure 6B). The DEPs between the control and PEDV groups were mainly enriched in biological processes related to lipid metabolic process (e.g., long-chain fatty acid metabolic process, fatty acid beta-oxidation) (Figure 6D). KEGG analysis showed that lipid metabolism (fatty acid degradation, PPAR signaling pathway, fatty acid metabolism) and amino acid metabolism (arginine and proline metabolism) were significantly enriched (Figure 6F). Reactome analysis suggested that metabolism, such as metabolism of amino acids and derivatives, were significantly enriched (Figure 6H). In addition, twenty core proteins were identified as the hub proteins according to the PPI network (Figure S5).

**SUPPLEMENTARY DATA**


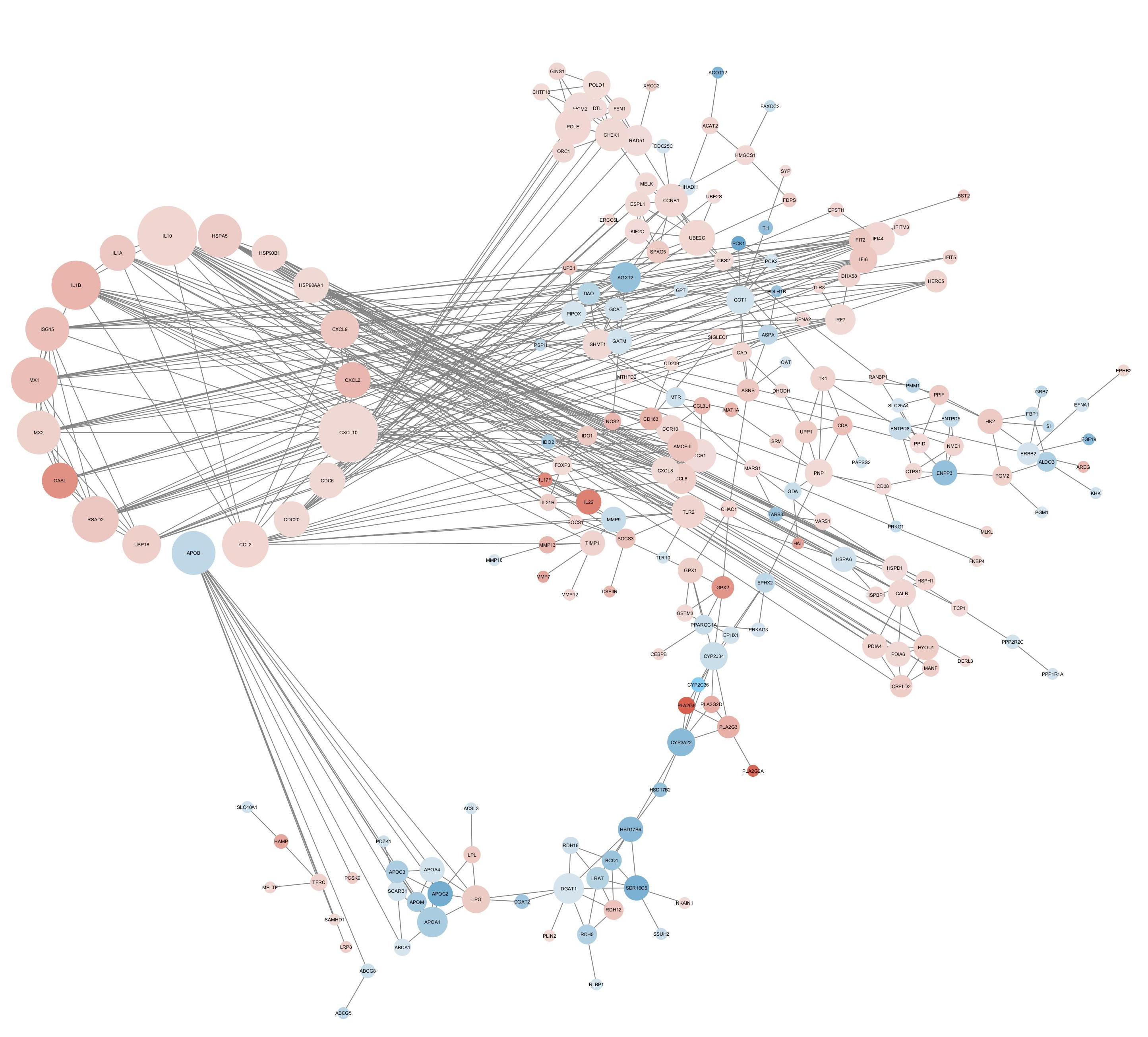


Figure S1. Hub genes identified in trial 1 through interactive analysis using the STRING database. Color-coded dots represent gene regulation status (red for up-regulation, blue for down-regulation), with dot size corresponding to core protein degree.


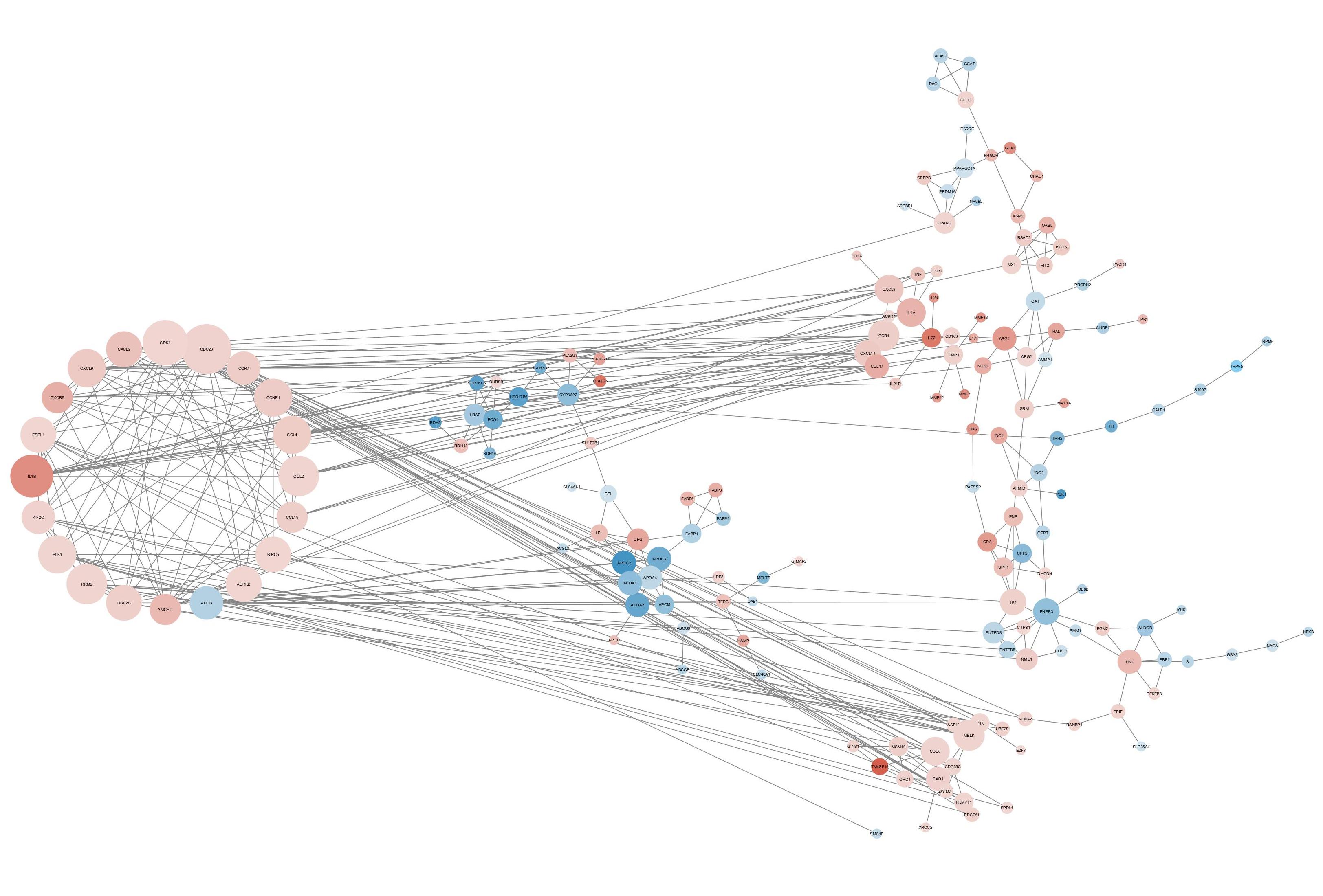


Figure S2. Hub genes identified in trial 2 through interactive analysis using the STRING database. Color-coded dots represent gene regulation status (red for up-regulation, blue for down-regulation), with dot size corresponding to core protein degree.


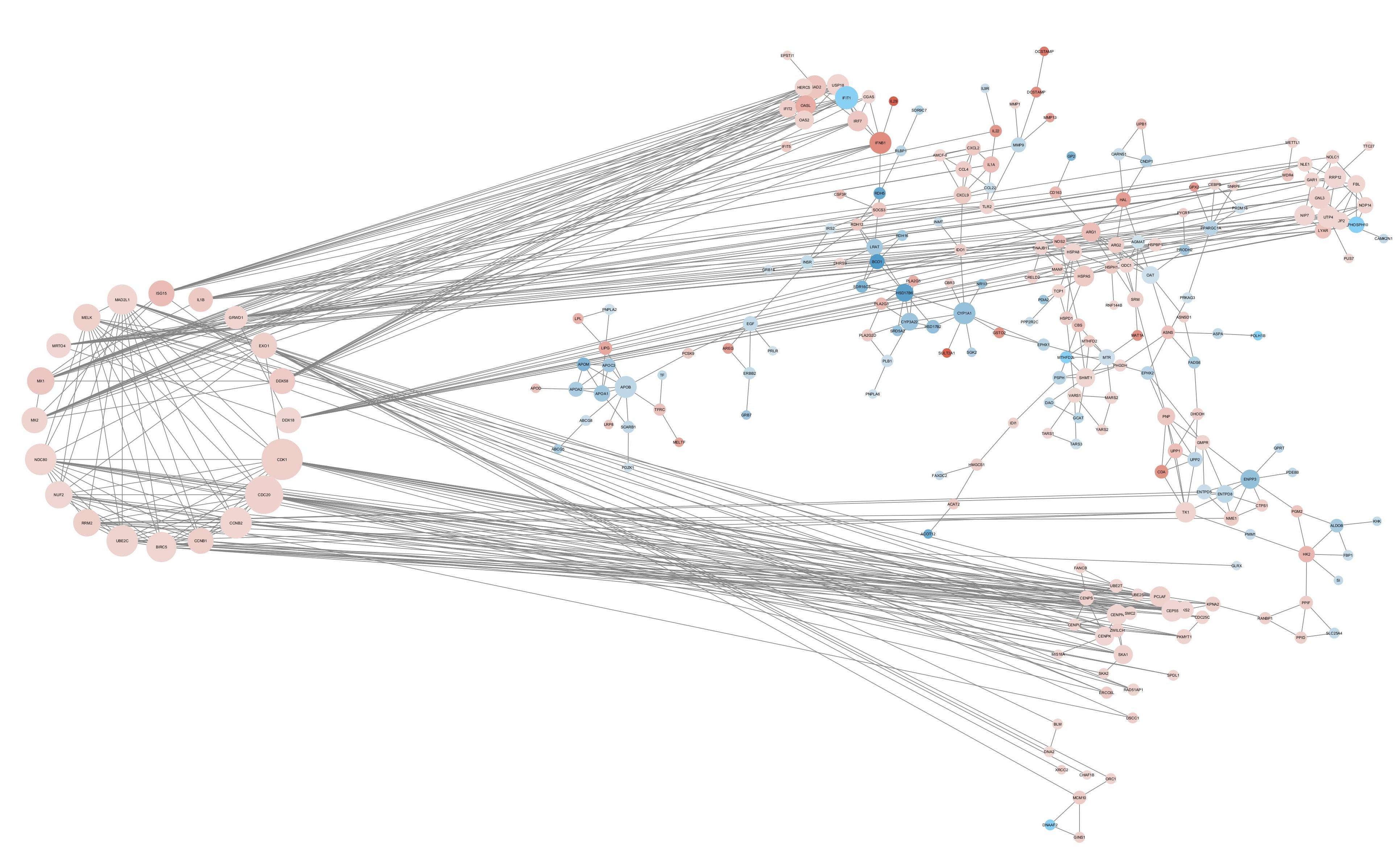


Figure S3. Hub genes identified in trial 3 through interactive analysis using the STRING database. Color-coded dots represent gene regulation status (red for up-regulation, blue for down-regulation), with dot size corresponding to core protein degree.


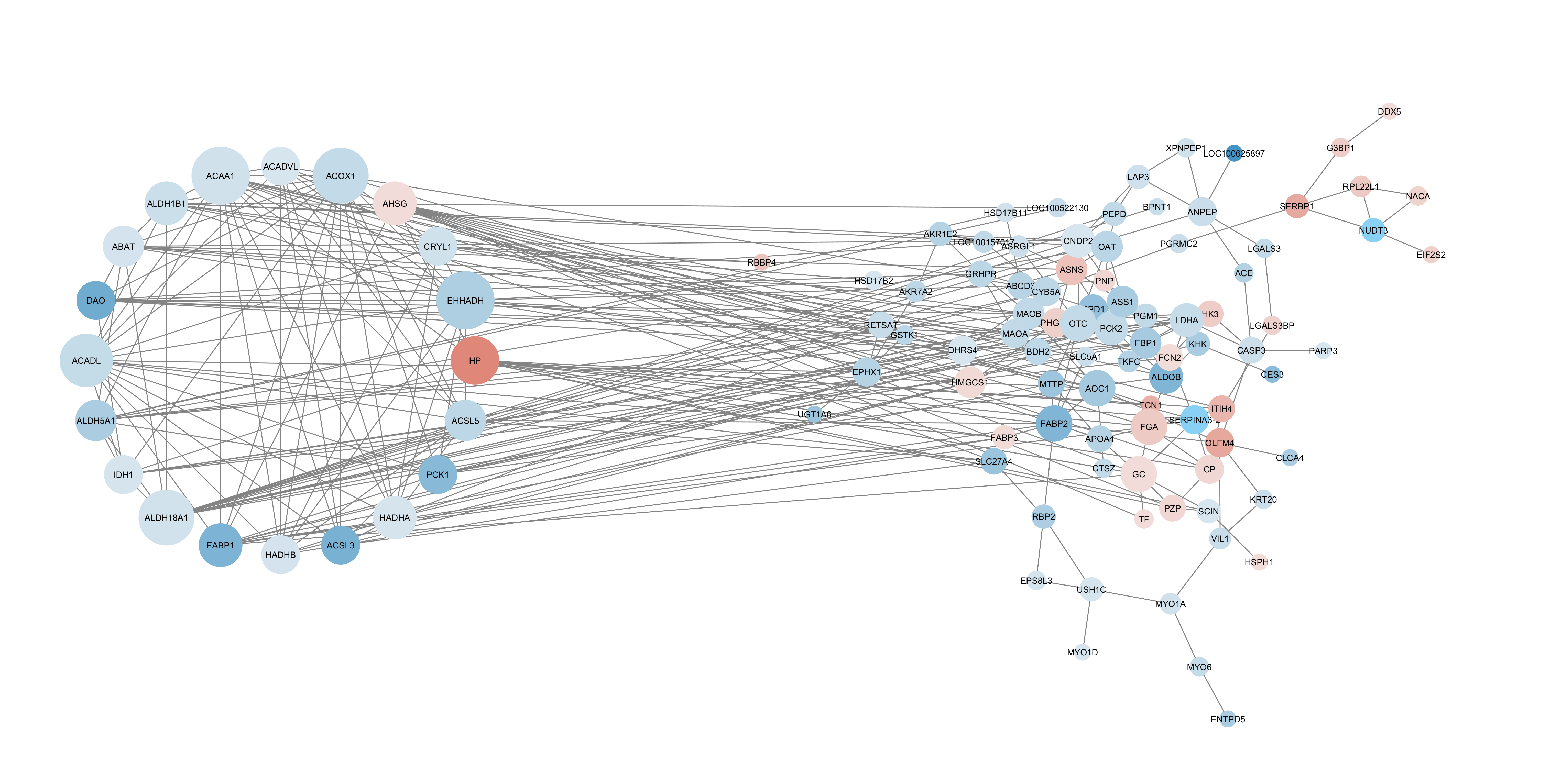


Figure S4. Hub genes identified in trial 4 through interactive analysis using the STRING database. Color-coded dots represent gene regulation status (red for up-regulation, blue for down-regulation), with dot size corresponding to core protein degree.


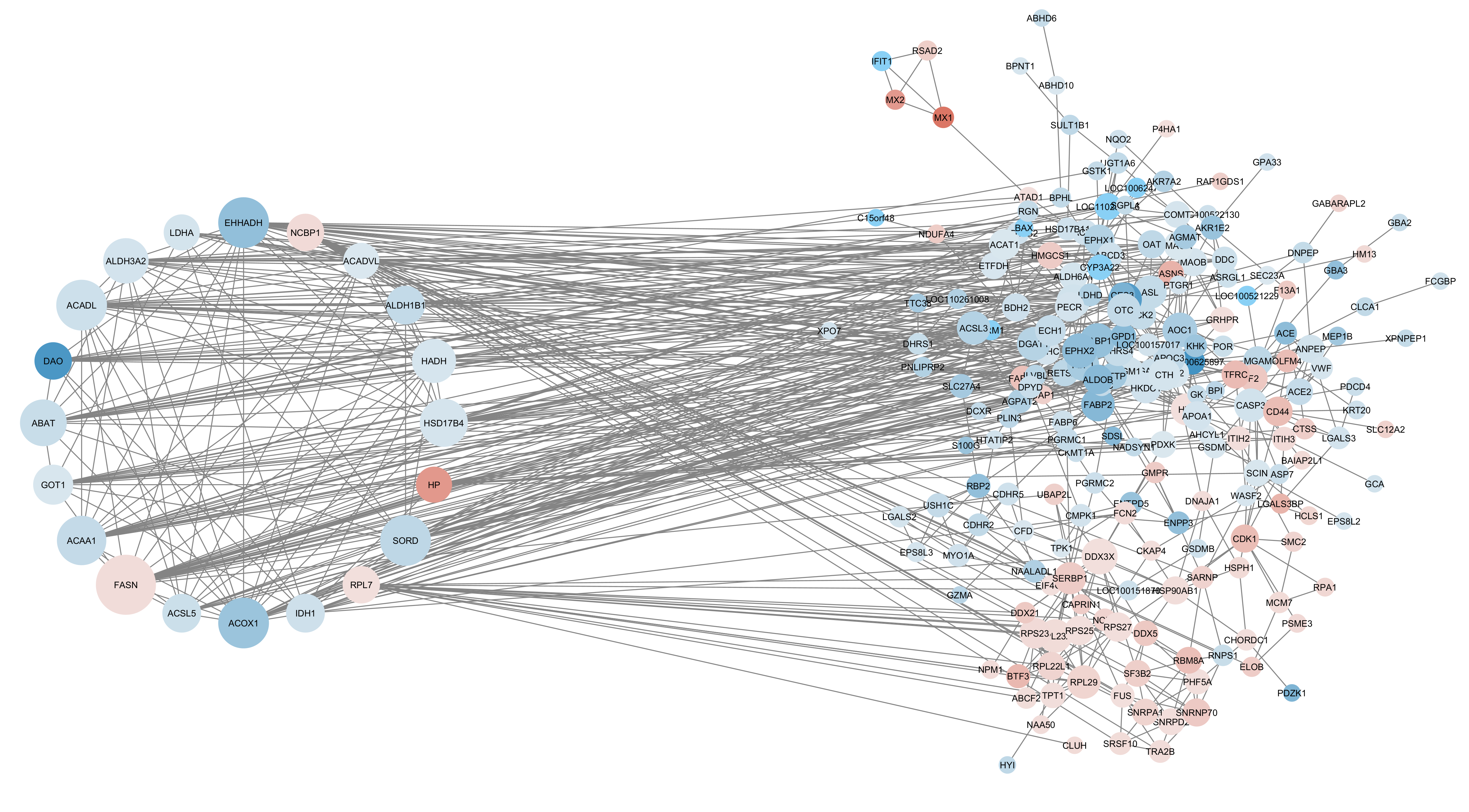


Figure S5. Hub genes identified in trial 5 through interactive analysis using the STRING database. Color-coded dots represent gene regulation status (red for up-regulation, blue for down-regulation), with dot size corresponding to core protein degree.
